# Foliar GR24 and γ-aminobutyric acid modulate redox signaling, root-system architecture, and mineral homeostasis in tellurite-stressed wheat (*Triticum aestivum* L.)

**DOI:** 10.1080/15592324.2026.2728143

**Published:** 2026-09-11

**Authors:** Imran Haider, Sana ur Rehman, Zafar Iqbal, Aqib Mahmood, Azatullah Zaheer, Muhammad Yousaf Nadeem, Muhammad Mubushar, Muhammad Usman Tahir

**Affiliations:** a National Research Center of Intercropping, The Islamia University of Bahawalpur, Bahawalpur, Pakistan; b Department of Biology, Nakhchivan State University, Nakhchivan, Azerbaijan; c Department of Business Administration, Faculty of Economics, Salam University, Kabul, Afghanistan; d College of Agriculture, Nanjing Agricultural University, Jiangsu, China; e Department of Plant Production, College of Food and Agriculture Sciences, King Saud University, Riyadh, Saudi Arabia

**Keywords:** γ-aminobutyric acid, strigolactone analogue, ROS-NO signaling, root plasticity, tellurium, glutathione redox state, mineral selectivity

## Abstract

Tellurite (TeO₃^2−^) is a phytotoxic tellurium (Te) species that disrupts cellular redox homeostasis, mineral nutrition, and root development. However, the signaling events underlying tellurite toxicity and its mitigation in cereals remain poorly understood. This study examined whether foliar application of GR24, a synthetic strigolactone analog, and *γ*-aminobutyric acid (GABA), individually or jointly, modulates early root signaling and downstream physiological acclimation in wheat (*Triticum aestivum* L.) exposed to tellurite. A 2 × 4 factorial experiment was conducted using two tellurite conditions and four foliar treatments: solvent control, GR24, GABA, and GR24 + GABA, with five replicate pots. Root reactive oxygen species (ROS), nitric oxide (NO), and calcium (Ca^2^⁺) signals were monitored for 72 h following the first foliar application. Redox homeostasis, plasma-membrane functioning, root-system architecture, rhizosphere properties, elemental composition, mineral/Te selectivity, and subcellular Te partitioning were subsequently assessed. Both signaling compounds alleviated tellurite-induced injury, with their combined application producing the strongest response by improving redox balance, membrane energization, mineral selectivity, root-system development, and root cell-wall Te retention. Relative to tellurite alone, GR24 + GABA restored total root length from 306 to 536 cm plant^−1^ (a 75.2% increase) and reduced the root-to-shoot Te translocation factor from 0.409 to 0.123 (a 69.9% decrease). These findings suggest that GR24 and GABA interactively improve tellurite tolerance by reshaping early redox-NO-Ca^2^⁺ signaling, restoring membrane energization, and coordinating selective nutrient acquisition with root-level Te sequestration.

## Graphical abstract

**Figure:**
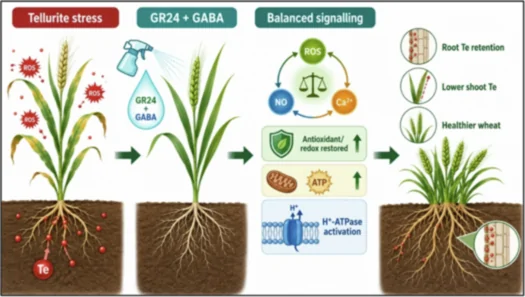

## 1. Introduction

Tellurium occupies an unusual position among trace elements. It is scarce in the lithosphere, yet its use in cadmium telluride photovoltaics, thermoelectric devices, alloying and vulcanization has concentrated it in specific industrial waste streams, and the accelerating turnover of solar and electronic hardware is expected to increase its release into agricultural soils over the coming decades.^1^ ^,^ ^2^ Unlike cadmium or arsenic, tellurium has no established regulatory threshold in most agricultural frameworks, and baseline data for cereal-growing soils are sparse.^3^ The reported background concentrations are on the order of 0.001–0.01 mg kg^−1^, rising to approximately 0.17  mg kg^−1^ in soils around electronic-waste processing sites,^3^ so tellurium enrichment is currently a localized rather than a widespread agricultural problem. The oxyanion tellurite, TeO₃^2-^, is the species most often encountered in oxic soils and is markedly more toxic to microorganisms and plants than elemental tellurium or tellurate.^4^ ^,^ ^5^ The toxicity of tellurite is chemically distinctive. Because the oxyanion is a strong oxidant that reacts readily with cellular thiols, it depletes reduced glutathione, generates superoxide during redox cycling, and can be reduced intracellularly to elemental tellurium, forming deposits that disturb membrane and organelle function.^6^ ^,^ ^7^ This thiol-directed mode of action distinguishes tellurite from metals that act primarily by displacing cofactors, and it predicts that the glutathione pool should be an early and sensitive reporter of tellurite exposure.^8^ In bacteria, tellurite resistance is closely tied to thiol and redox metabolism;^9^ in yeast, sulfate assimilation has been shown to mediate both tellurite reduction and toxicity;^10^ the equivalent picture in higher plants is far less developed, and in wheat it is almost absent.

Available evidence indicates that tellurite exposure inhibits root elongation more sharply than shoot growth, disturbs membrane integrity, and interferes with the acquisition of sulfur and phosphorus-an expected consequence of the chemical similarity between tellurite and the sulfate and phosphate oxyanions, which share transport routes at the root plasma membrane.^10^ ^,^ ^11^ Because tellurite resembles both oxyanions, the sulfur and phosphorus status of the plant may be both a determinant of tellurium uptake and a casualty of it; the relative contribution of the sulfate and phosphate routes has not been resolved in any higher plant.^12^ Whether tellurite additionally acts as a signal that the plant mounts a structured, time-resolved perception response rather than simply accumulating damage has not been established for any cereal. Reactive oxygen species occupy a dual role in plant stress physiology that is now well established: at low and spatially controlled concentrations, they act as second messengers coordinating acclimation, while unrestrained accumulation causes oxidative injury.^13^ ^,^ ^14^ Hydrogen peroxide is the principal mobile species in this signaling role, owing to its relative stability and its ability to cross membranes through aquaporins, whereas superoxide produced at the apoplastic face by respiratory burst oxidase homologs acts more locally.^15^ ^,^ ^16^ The distinction matters for the interpretation of any stress experiment: a rise in H₂O₂ is not, on its own, evidence of damage, and the temporal profile of that rise carries more information than its magnitude at a single time point.^17^

Nitric oxide interacts with this system at several levels. It modifies protein function through S-nitrosylation, participates in the regulation of antioxidant enzymes, and shows characteristic early transients under metal and metalloid stress.^18^ ^,^ ^19^ The relative timing of the ROS and NO signals influences whether the downstream outcome is acclimation or programmed cell death, since the two converge on overlapping targets, including components of the glutathione system.^20^ Cytosolic calcium transients constitute the third element of this early signaling triad. Elevations in cytosolic Ca^2^⁺ are among the earliest measurable responses to a wide range of abiotic stimuli and are decoded by calcium-dependent protein kinases and related sensors that feed back onto NADPH oxidase activity, establishing a self-reinforcing ROS-Ca^2^⁺ loop.^21^ ^,^ ^22^ In roots, these events are spatially structured, being concentrated in the apex and elongation zones, where growth responses are determined.^23^ It follows that a description of tellurite perception in wheat requires measurements that are resolved in both time and space, rather than bulk determinations at harvest. Strigolactones were characterized first as rhizosphere signals and subsequently as hormones that regulate shoot branching, and their role in shaping root-system architecture is now well documented.^24^ The direction of that effect is conditional. Under phosphate sufficiency, applied GR24 suppresses lateral root primordium development and reduces lateral root density in Arabidopsis, an inhibition that weakens under phosphate limitation;^25^ the same suppression has been reported in Arabidopsis biosynthesis mutants but not in signaling mutants.^26^ Strigolactone signaling promotes root hair elongation more consistently and is itself strongly induced by phosphate and nitrogen limitation, which positions the pathway as an integrator of nutrient availability and root foraging behavior.^25^ ^,^ ^26^ The synthetic analog GR24 reproduces many of these responses and is the standard experimental tool in the field. Two features make GR24 an attractive candidate for tellurite stress specifically. Strigolactone signaling has been repeatedly associated with altered redox status and antioxidant capacity under abiotic stress, although whether this reflects a direct action on redox homeostasis or a downstream consequence of improved growth remains contested.^27^ Strigolactones move in the transpiration stream and participate in shoot-to-root communication, so a foliar application can plausibly influence root behavior without direct contact between the compound and the contaminated soil – an important practical consideration where a soil-applied amendment would itself alter tellurium speciation.^28^

*γ*-Aminobutyric acid accumulates rapidly in plant tissues under a wide range of stresses, and its status has shifted over the past decade from that of a metabolic by-product to that of a signaling metabolite in its own right.^29^ GABA influences ROS homeostasis, contributes carbon and nitrogen to the tricarboxylic acid cycle through the GABA shunt when the cycle is disrupted, and has been implicated in the regulation of anion transport at the plasma membrane, providing a plausible mechanistic connection to ion selectivity and membrane energization.^30^ GABA modulates stomatal behavior and root growth, and it acts on membrane transport processes that govern the balance between the uptake of essential nutrients and that of toxic analogs. GABA is a negative regulator of the aluminum-activated malate transporter TaALMT1 and inhibits malate efflux from the root,^30^ ^,^ ^31^ so its effect on rhizosphere organic acid chemistry is expected to be restrictive rather than stimulatory. Under tellurite stress, where the central physiological problem is the plant's inability to discriminate between a toxic oxyanion and the sulfate and phosphate it resembles, a treatment acting on transport selectivity is mechanistically distinct from one acting on antioxidant capacity alone. This raises the possibility that GR24 and GABA are complementary rather than redundant: GR24 acts predominantly on root architecture and foraging behavior, GABA on membrane transport and redox buffering.

No previous study has examined the effects of foliar GR24 or GABA on wheat exposed to tellurite stress or resolved the early signaling response to tellurite in cereals over time. Therefore, the present study aimed to characterize the time-resolved ROS-NO-Ca^2^⁺ signaling response induced by tellurite in wheat roots and distinguish it from progressive oxidative injury. We also aimed to determine whether foliar GR24 and GABA modify the amplitude and duration of these signals, promote adaptive changes in root-system architecture, root-hair development, and gravitropic responsiveness, restore membrane energization, and improve mineral selectivity against tellurium. Finally, this study evaluated treatment-induced changes in tissue accumulation, root-to-shoot translocation, and subcellular partitioning of tellurium.

The present study provides the first integrated assessment of the individual and combined effects of GR24 and GABA on tellurite-stressed wheat. This study is also the first cereal study to integrate time-resolved root ROS-NO-Ca^2^⁺ signaling with subsequent changes in the cellular redox status, membrane energization, root-system plasticity, mineral/Te selectivity, and subcellular Te partitioning. Previous studies have predominantly examined GR24 or GABA separately under more commonly investigated abiotic stresses, such as drought, salinity, and cadmium toxicity, with an emphasis mainly on endpoint antioxidant responses, photosynthetic performance, and plant growth. The novelty of the present investigation, therefore, extends beyond the simple co-application of two established stress regulators. Using a factorial design, we tested whether their potentially complementary physiological functions-GR24-associated regulation of stress signaling and root development, and GABA-mediated regulation of redox balance, energy metabolism, and anion transport – could produce a broader and more effective response than either compound applied individually. Accordingly, this study characterized the time-dependent ROS-NO-Ca^2^⁺ response induced by tellurite in wheat roots, distinguished early signaling events from progressive oxidative injury, and evaluated whether combined GR24 and GABA application provided greater physiological recovery than the individual treatments. The investigation further linked early signal modulation with root-system architecture, root-hair development, gravitropic responsiveness, plasma-membrane proton-translocating adenosine triphosphatase (H⁺-ATPase) activity, mineral selectivity, tissue Te accumulation, root-to-shoot translocation, and subcellular Te sequestration.

## 2. Materials and methods

### 2.1. Plant material and growth conditions

Seeds of wheat (*Triticum aestivum* L. cv. Galaxy-2013) were obtained from the Regional Agricultural Research Institute, Bahawalpur, Punjab, Pakistan. Uniform and healthy seeds were surface-sterilized with 5% sodium hypochlorite for 5 min, rinsed thoroughly with deionized water, and sown in plastic pots containing 8 kg of air-dried and 2-mm-sieved soil. Ten seeds were sown in each pot and thinned to five uniform plants at 10 d after sowing (DAS). The experiment was conducted during the 2022–2023 winter cropping season in a wire-house at The Islamia University of Bahawalpur, Bahawalpur, Pakistan (29°23′ N, 71°41′ E; 117 m above sea level). Seeds were sown in mid-November 2022, and final physiological and biochemical measurements were performed at 75 DAS. The mean day/night temperature was approximately 25 ± 3/15 ± 3 °C, the relative humidity ranged from 55% to 65%, and the natural photoperiod varied from 10.2 to 11.0 h. The photosynthetic photon flux density around solar noon averaged approximately 1,000  µmol photons m^−2^ s^−1^ on clear days. The soil moisture was maintained at approximately 70% field capacity by weighing the pots every 48 h and replacing the water lost through evapotranspiration. Field-capacity moisture was calculated as:

$$\text{Field}-\text{capacity moisture}(\%)=\frac{W_{\mathrm{FC}}-W_{\mathrm{dry}}}{W_{\mathrm{dry}}}\times 100$$

where W_FC_ is the soil mass at field capacity, and W_dry_ is the oven-dry soil mass. Pots were completely re-randomized every 7 d to minimize positional effects associated with light, temperature, and air circulation. All treatments received identical agronomic management throughout the experiment.

### 2.2. Soil physicochemical characterization

Bulk surface soil was collected from the 0–30 cm depth of the experimental farm at The Islamia University of Bahawalpur, Pakistan. The soil was air-dried under shade, thoroughly homogenized, and passed through a 2-mm sieve. Three independent composite samples were used to characterize the initial physicochemical properties of the substrate. The particle size distribution was determined using the hydrometer method.^32^ The soil pH and electrical conductivity were measured in a 1:2.5 (w/v) soil-to-water suspension. The organic matter content was determined using the Walkley-Black wet-oxidation method.^33^ The available nitrogen content was quantified by the alkaline potassium-permanganate method, available phosphorus by sodium-bicarbonate extraction,^34^ the exchangeable potassium by neutral ammonium-acetate extraction, and the available sulfur by calcium-chloride extraction. DTPA-extractable Fe and Zn were quantified following Lindsay and Norvell.^35^ Background soil Te was determined by inductively coupled plasma mass spectrometry (ICP-MS) following acid digestion, while field capacity was measured gravimetrically. The substrate was classified as sandy loam, containing 56.3% sand, 33.2% silt, and 10.5% clay. It was slightly alkaline, with a pH of 7.42, and non-saline, with an electrical conductivity of 1.36 dS m^−1^. The organic matter and available nitrogen contents were 0.83% and 42.6  mg kg^−1^, respectively. The Olsen-extractable phosphorus, exchangeable potassium, and available sulfur contents were 7.82, 112.0, and 8.64  mg kg^−1^, respectively. DTPA-extractable Fe and Zn contents were 4.72 and 0.68  mg kg^−1^, respectively. The background Te was 0.03  mg kg^−1^, whereas the gravimetrically determined field capacity was 24.8%.

### 2.3. Tellurite treatment

Sodium tellurite (Na₂TeO₃; purity ≥99%; Sigma-Aldrich) was used as the Te source. A preliminary dose‒response experiment was conducted using 0, 15, 30, 60, 90, and 120  mg Te kg^−1^ dry soil. A concentration of 60  mg Te kg^−1^ was selected because it induced measurable stress without causing extensive tissue necrosis or plant mortality. This concentration is well above the reported background and contaminated-soil values^3^ and was chosen as a mechanistic screening dose capable of resolving early signaling events within a single growing season, rather than as an environmentally representative exposure. The amount of sodium tellurite needed to provide the required elemental Te concentration was calculated on a molecular-mass basis, giving 104.2 mg Na₂TeO₃ kg^−1^ soil or 833.5 mg Na₂TeO₃ per 8-kg pot. Sodium tellurite was applied uniformly before sowing, and the treated soil was equilibrated at 70% field capacity for 14 d. The control soil received the same volume of deionized water and underwent identical mixing and equilibration.

### 2.4. Foliar application of GR24 and GABA

The synthetic strigolactone analog *rac*-GR24 (Chiralix B.V., Nijmegen) was applied at 1  µM. GABA (*γ*-aminobutyric acid; purity ≥99%; Sigma-Aldrich) was applied at 1  mM. The combined treatment contained 1 µM GR24 and 1 mM GABA. These concentrations were selected from preliminary screening and previously reported physiologically effective treatments.^36^ ^,^ ^37^ Foliar GR24 at 1 µM has been shown to reduce metal uptake, enhance nitric oxide signaling, and activate the ascorbate‒glutathione cycle in barley under cadmium stress,^36^ whereas 1.0 mM GABA was the most effective concentration in wheat seedlings under drought.^37^ The final acetone concentration was maintained at 0.1% (v/v), and all spray solutions contained 0.01% Tween-20. The same acetone and surfactant concentrations were included in the solvent control. Foliar treatments were applied at 25, 35, and 45 DAS during the early morning. Approximately 20  mL of solution was applied per plant until incipient run-off. The soil surface was covered during spraying to prevent the foliar treatments from reaching the rhizosphere.

### 2.5. Experimental design and treatment structure

The main pot experiment followed a 2 × 4 factorial completely randomized design. Factor A consisted of two soil tellurite levels: 0 and 60  mg Te kg^−1^ soil. Factor B consisted of four foliar treatments: solvent control, 1 µM GR24, 1 mM GABA, and 1 µM GR24 +1 mM GABA (Table 1). Five independent pots were maintained per treatment, resulting in 40 pots. Each pot constituted one biological replicate and experimental unit. Measurements obtained from multiple plants, leaves, or roots within a pot were treated as subsamples and averaged before statistical analysis. Separate experimental sets were established for destructive signaling measurements, rhizobox observations, and gravitropic assays. The study used five independent biological replicates per treatment rather than biological triplicates. The unstressed solvent-control treatment and the tellurite-stressed solvent control each comprised five independent pots (*n* = 5).

**Table 1.** Factorial treatment structure.

Code	Tellurite level	Foliar treatment
C	0 mg Te kg^−1^	Solvent control
GR24	0 mg Te kg^−1^	GR24 at 1 µM
GABA	0 mg Te kg^−1^	GABA at 1 mM
GR24 + GABA	0 mg Te kg^−1^	GR24 at 1 µM + GABA at 1 mM
Te	60 mg Te kg^−1^	Solvent control
Te + GR24	60 mg Te kg^−1^	GR24 at 1 µM
Te + GABA	60 mg Te kg^−1^	GABA at 1 mM
Te + GR24 + GABA	60 mg Te kg^−1^	GR24 at 1 µM + GABA at 1 mM

### 2.6. Temporal signaling measurements

Root tissue was sampled at 0, 6, 24, and 72 h relative to the first foliar application at 25 DAS. The 0-h sample was collected immediately before spraying and represented the pre-application baseline. Therefore, the time-course data described the responses after foliar application in plants already growing in tellurite-treated or untreated soil. Hydrogen peroxide was quantified by potassium-iodide oxidation,^38^ whereas the superoxide-production rate was determined by hydroxylamine oxidation.^39^ NO-related nitrite equivalents were measured by the Griess-reagent method.^40^ NADPH oxidase activity was determined in a plasma-membrane-enriched fraction using superoxide-dependent nitroblue-tetrazolium reduction with appropriate superoxide-dismutase controls.^41^ H₂O₂, O₂•-, NO and NADPH oxidase activity were expressed as µmol H₂O₂ g^−1^ fresh weight, nmol O₂•- g^−1^ fresh weight min^−1^, nmol NO equivalents g^−1^ fresh weight and nmol O₂•- min^−1^ mg^−1^ protein, respectively. Five independent pots were analyzed per treatment at each sampling time.

### 2.7. Histochemical and fluorescence localization

Hydrogen peroxide and superoxide were localized in the root tips by 3,3′-diaminobenzidine and nitroblue-tetrazolium staining, respectively.^42^ ^,^ ^43^ Total ROS-associated fluorescence was assessed using H₂DCFDA, whereas NO-associated fluorescence was determined using DAF-FM DA.^44^ Root Ca^2^⁺ status was assessed using a single-wavelength Ca^2^⁺-responsive probe following Kiegle et al.^45^ Because a calibrated ratiometric indicator was not used, Ca^2^⁺ results were reported as relative Ca^2^⁺-associated fluorescence rather than absolute cytosolic Ca^2^⁺ concentration. The fluorescence and staining intensities were quantified in ImageJ within the root apex, elongation zone, and lateral-root initiation zone. Identical imaging settings were maintained for all the treatments, and probe-free roots were used for background correction. Six roots distributed across five independent pots were examined per treatment.

### 2.8. Root-system architecture, spatial exploration, and behavior

#### 2.8.1. Whole-root architecture

Whole-root architecture, including total root length, surface area, volume, mean diameter, root-tip number, lateral-root number, and fine-root length, was determined through digital root-image analysis following Himmelbauer et al.^46^ Identical image-acquisition and software settings were maintained across treatments. Specific root length was calculated as total root length (m) per unit root dry mass (g). Lateral-root density was expressed as the number of lateral roots per centimeter of the main root axis. Measurements from plants belonging to the same pot were averaged to retain the pot as the experimental unit.

#### 2.8.2. Spatial root exploration

Spatial root distribution was evaluated in transparent-front rhizoboxes according to the phenotyping approach described by Nagel et al.^47^ Maximum rooting depth, horizontal spread, seminal-root angle, visible root length, and root convex-hull area were obtained from calibrated images. The root exploration index represented the convex-hull area occupied per unit visible root length. Four independent rhizoboxes were examined per treatment.

#### 2.8.3. Root-hair traits

Root-hair length, root-hair density, and root-hair-zone length were determined microscopically following Bates and Lynch.^48^ Six roots distributed across five independent pots were examined per treatment, and multiple observations within the same pot were averaged before statistical analysis.

#### 2.8.4. Gravitropic responsiveness

Root gravitropic behavior was assessed in vertical growth pouches using an image-based gravistimulation method.^49^ Tellurite was supplied at 0 or 0.47  mM Te, a concentration equivalent to 60 mg Te L^−1^ and therefore matched on a mass basis to the 60 mg Te kg-1 soil dose used in the pot experiment; shoots received the four designated foliar treatments. The growth-pouch assay was considered a companion mechanistic experiment rather than an exact reproduction of soil Te exposure. Root-tip curvature, reorientation, final gravitropic angle, and root elongation were measured at 0, 2, 4, and 6 h after rotating the growth pouches through 90°. Fifteen seedlings per treatment were assessed across three independent experimental runs.

#### 2.8.5. Root vitality and cell viability

Root metabolic vitality was determined by triphenyltetrazolium-chloride reduction,^50^ whereas root-tip cell injury was assessed by Evans blue uptake.^51^ The results are expressed as µg triphenyl formazan g^−1^ fresh weight h^−1^ and relative Evans blue uptake, respectively. Four independent pots were analyzed per treatment.

### 2.9. Oxidative damage and antioxidant defense

#### 2.9.1. Oxidative and membrane damage

Malondialdehyde was measured by the thiobarbituric-acid-reactive-substances method,^52^ and the protein carbonyl content was determined by 2,4-dinitrophenylhydrazine derivatization.^53^ Electrolyte leakage was calculated as (EC₁/EC₂) × 100, where EC₁ is the conductivity after initial incubation and EC₂ is the conductivity after complete tissue disruption and cooling. The membrane stability index was determined independently as a two-bath conductivity measurement following Sairam et al.^54^ Leaf discs were held at 40 °C for 30 min (C₁) and at 100 °C for 10 min (C₂), and the MSI was calculated as [1 - (C₁/C₂)] × 100. MSI and electrolyte leakage are therefore separate determinations on separate tissue samples and are not algebraic complements of one another.

#### 2.9.2. Antioxidant enzymes

Superoxide dismutase activity was determined according to Giannopolitis and Ries,^55^ catalase according to Aebi,^56^ ascorbate peroxidase according to Nakano and Asada^57^ and glutathione reductase according to Foyer and Halliwell.^58^ Enzyme activities were normalized to soluble-protein concentration and expressed in the appropriate activity units.

#### 2.9.3. Ascorbate-glutathione and energy status

Reduced and oxidized ascorbate were determined following Law et al.^59^ whereas GSH and GSSG were measured by the enzymatic recycling method of Griffith.^60^ The AsA/DHA and GSH/GSSG ratios were calculated from replicate-level concentrations. The glutathione half-cell reduction potential was estimated according to Schafer and Buettner^61^ as E_h_ = E°′ - (RT/2F) ln([GSH]^2^/[GSSG]), where E°′ is the standard potential adjusted for pH, R is the gas constant, T is the absolute temperature, and F is the Faraday constant. NADPH and NADP⁺ were determined by enzymatic cycling,^62^ whereas ATP was quantified by luciferin-luciferase bioluminescence.^63^

### 2.10. Plasma-membrane function and rhizosphere chemistry

Plasma-membrane H⁺-ATPase activity was determined as vanadate-sensitive inorganic-phosphate release following Palmgren,^64^ while root proton extrusion was assessed from the acidification of an initially unbuffered bathing solution.^65^ Rhizosphere pH and redox potential were measured using calibrated electrodes. Acid-phosphatase activity was determined with *p*-nitrophenyl phosphate,^66^ and available rhizosphere Te was quantified by ICP-MS. Citrate, malate, and oxalate contents in the root exudates were determined by HPLC following Neumann and Römheld.^67^ Plant-free blanks were included, and organic-acid release was expressed per unit root dry mass and collection time.

### 2.11. Mineral nutrition and tellurium determination

The roots were desorbed in ice-cold 10 mM CaCl₂ to minimize surface-bound Te. Dried root and shoot tissues were digested with trace-metal-grade nitric acid and hydrogen peroxide. Te, Fe, and Zn were determined by ICP-MS, whereas S, P, K, and Ca were quantified by ICP-OES following validated plant elemental-analysis procedures.^68^ Digestion blanks, continuing calibration standards, analytical duplicates, and NIST SRM 1567b wheat flour were included for quality assurance. A reference material recovery of 85%–115% and a duplicate relative standard deviation below 10% were considered acceptable. Instrument-specific detection and quantification limits were reported in the Supplementary Material. Mineral-to-Te selectivity ratios were calculated on a molar basis as (C_mineral_/M_mineral_) ÷ (C_Te_/M_Te_). The root and shoot bioaccumulation factors were calculated relative to the measured soil Te concentration. Te exclusion efficiency was determined relative to the tellurite-stressed solvent control. The root-to-shoot translocation factor was calculated as shoot Te divided by root Te. All indices were calculated from individual biological replicates rather than from treatment means. Values below the quantification limit were treated as censored observations and were not replaced with the detection limit when calculating concentration ratios. The root tissue was separated into cell-wall, organelle, and soluble fractions by differential centrifugation following the methods of Weigel and Jäger.^69^ Te in each fraction was quantified by ICP-MS and expressed as a percentage of total recovered Te. Fractionation recovery was verified against an unfractionated root aliquot, and samples outside the 80%–120% recovery range were reanalyzed. The soluble fraction was not interpreted as exclusively cytosolic because it could include cytosolic and vacuolar constituents (Table 2).

**Table 2.** Principal parameters, sampling stages, and cited methods.

Parameter group	Principal variables	Sampling stage	Method citation
Early signaling	H₂O₂, O₂•-, NO, NADPH oxidase	0, 6, 24, and 72 h	Lindsay and Norvell [35]; Qiu et al. [36]; Zhao et al. [37]; Velikova et al. [38]
Histochemical imaging	DAB, NBT, H₂DCFDA, DAF-FM DA	Selected signaling stage	Elstner and Heupel [39]; Zhou et al. [40]; Sagi and Fluhr [41]
Root architecture	Root length, surface area, volume, and diameter	75 DAS	Dunand et al. [43]
Spatial root behavior	Rooting depth, spread, angle, and convex-hull area	Rhizobox harvest	Rodríguez-Serrano et al. [44]
Root hairs and gravitropism	Hair length and density; gravitropic curvature	Selected stages	Kiegle et al. [45]; Himmelbauer et al. [46]
Oxidative damage	MDA, electrolyte leakage, protein carbonyls	75 DAS	Grabov et al. [49]; Steponkus and Lanphear [50]
Antioxidant defense	SOD, CAT, APX, and GR	75 DAS	Heath and Packer [52]; Levine et al. [53]; Sairam et al. [54]; Giannopolitis and Ries [55]
Redox and energy status	AsA/DHA, GSH/GSSG, NADPH/NADP⁺, and ATP	75 DAS	Nakano and Asada [57] ; Aebi [56]; Law et al. [59]
Membrane function	H⁺-ATPase and proton extrusion	75 DAS	Schafer and Buettner [61]; Queval and Noctor [62]
Rhizosphere chemistry	pH, redox potential, phosphatase, and organic acids	75 DAS	Lundin [63]; Palmgren [64]
Mineral and Te analysis	S, P, K, Ca, Fe, Zn, Te, and derived indices	75 DAS	Yan et al. [65]
Subcellular Te	Cell-wall, organelle, and soluble fractions	75 DAS	Tabatabai and Bremner [66]

### 2.12. Growth, photosynthetic performance, and water status

Plant growth and physiological characteristics were evaluated at the final physiological sampling stage. Plant height was measured from the soil surface to the tip of the uppermost fully expanded leaf using a graduated measuring scale. The total leaf area per plant was measured using a portable leaf-area meter (LI-3000C, LI-COR Biosciences). After harvest, the roots were carefully separated from the shoots and washed with deionized water to remove adhering soil particles. The root and shoot samples were placed in separate paper bags, oven-dried at 65 °C to a constant mass, and weighed using an analytical balance. The root-to-shoot ratio was calculated by dividing the root dry weight by the shoot dry weight. The net photosynthetic rate (Pn) and stomatal conductance (gs) were measured on the uppermost fully expanded, healthy leaf between 09:00 and 11:00 h using a portable open gas-exchange system (LI-6400XT, LI-COR Biosciences, Lincoln, NE, USA), following the steady-state gas-exchange principles described by von Caemmerer and Farquhar.^70^ During measurement, the photosynthetic photon flux density within the leaf chamber was maintained at 1200 µmol m^−2^ s^−1^ using the integrated red-blue light source. The reference CO₂ concentration was maintained at 400  µmol mol^−1^, the airflow rate at 500  µmol s^−1^, and the leaf-block temperature was 25 ± 1 °C. Measurements were recorded only after the Pn, gs, and intercellular CO₂ concentration had reached stable values.

Chlorophyll fluorescence parameters were measured on the same leaves using a pulse-amplitude-modulated fluorometer (PAM-2500, Heinz Walz GmbH, Effeltrich, Germany). The selected leaves were dark-adapted for 30 min using dark-adaptation clips. Minimum fluorescence (F₀) and maximum fluorescence (Fm) were measured in dark-adapted leaves, and variable fluorescence was calculated as Fv = Fm − F₀. The maximum quantum efficiency of photosystem II was calculated as Fv/Fm = (Fm − F₀)/Fm.^71^ The effective quantum yield of photosystem II (ΦPSII) was determined under actinic illumination according to Genty et al.^72^ whereas non-photochemical quenching was calculated as NPQ = (Fm − Fm′)/Fm′ following the fluorescence-quenching approach of Bilger and Björkman.^73^

Leaf relative water content (RWC) was determined from fresh mass (FW), turgid mass (TW), and dry mass (DW) following Barrs and Weatherley.^74^ Fresh mass was recorded immediately after sampling. The leaf segments were floated on deionized water for 4 h in darkness, gently blotted, and weighed to obtain the TW. The samples were subsequently oven-dried at 65 °C to a constant mass to determine the DW. The relative water content was calculated as follows:

$$\mathrm{RWC}\left(\%\right)=\frac{\mathrm{FW}-\mathrm{DW}}{\mathrm{TW}-\mathrm{DW}}\times 100$$

Five independent pots were evaluated per treatment, and each pot was considered one biological replicate.

### 2.13. Statistical analysis

Data are presented as the means ± standard deviation (SD). For the main pot experiment, each treatment comprised five independent biological replicates (*n* = 5 pots), including five unstressed solvent-control pots and five tellurite-stressed solvent-control pots. Individual plants, leaves, roots, or repeated analytical readings obtained from the same pot were treated as subsamples and averaged before analysis; they were not considered independent biological replicates. Endpoint variables were analyzed by two-way factorial analysis of variance (ANOVA), with tellurite treatment (0 and 60 mg Te kg^−1^) and foliar treatment (solvent control, GR24, GABA, and GR24 + GABA) as fixed factors, including their interaction. Time-resolved signaling variables were analyzed by three-way factorial ANOVA with tellurite, foliar treatment, and sampling time as fixed factors; independent pots sampled at each time point constituted the experimental units. Before ANOVA, residual normality and homogeneity of variance were assessed using the Shapiro‒Wilk test and Levene test, respectively, together with graphical inspection of residuals. When a significant omnibus F-test was obtained, treatment means were separated using Tukey honestly significant difference (HSD) test. Statistical significance was evaluated at *α* = 0.05; exact *p*-values were reported where available, with *p* < 0.05, *p* < 0.01, and *p* < 0.001 used to denote increasing levels of significance. Non-significant effects were identified as p ≥ 0.05. ANOVA and Tukey HSD tests were performed in Statistix version 8.1. Pearson correlation analysis, principal component analysis, and hierarchical cluster analysis were conducted in OriginPro 2021, and graphs were prepared using GraphPad Prism version 10.

## 3. Results

### 3.1. GR24 and GABA reshape early ROS-NO-Ca^2^⁺ signatures

**Figure 1.**
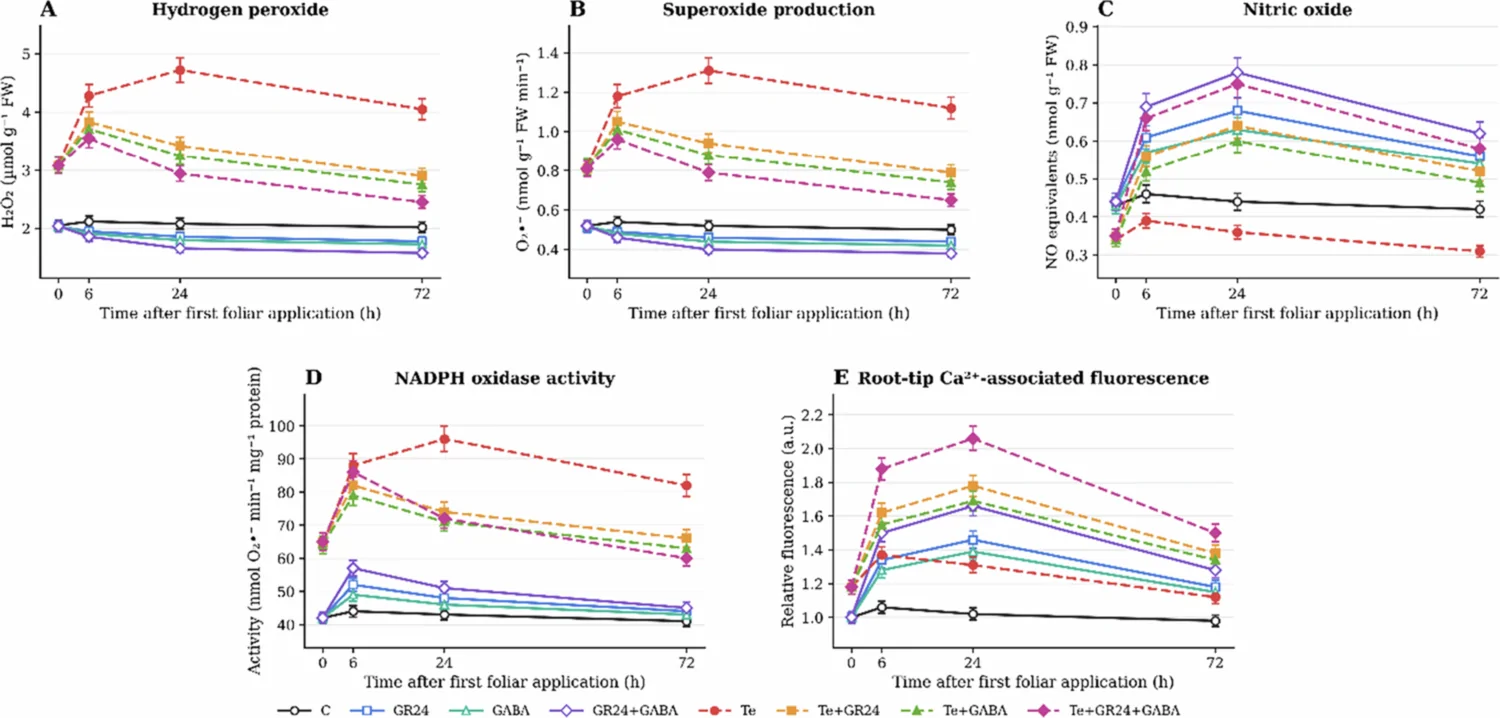
Temporal changes in early redox and calcium-associated signals in wheat following foliar GR24 and *γ*-aminobutyric acid (GABA) application under tellurite stress. (A) Hydrogen peroxide (H₂O₂) content; (B) superoxide (O₂•-)-production rate; (C) nitric oxide (NO) level; (D) nicotinamide adenine dinucleotide phosphate (NADPH) oxidase activity; and (E) root-tip calcium (Ca^2^⁺)-associated fluorescence. The treatments used were: C, untreated control; GR24, 1  µM GR24; GABA, 1  mM GABA; GR24 + GABA, combined treatment; Te, 60  mg tellurite kg^−1^ soil; and the corresponding Te + foliar-treatment combinations. Measurements were made at 0, 6, 24, and 72 h relative to the first foliar spray at 25 d after sowing. The values are the means ± standard deviation of five independent pots per treatment and sampling time.

**Figure 2.**
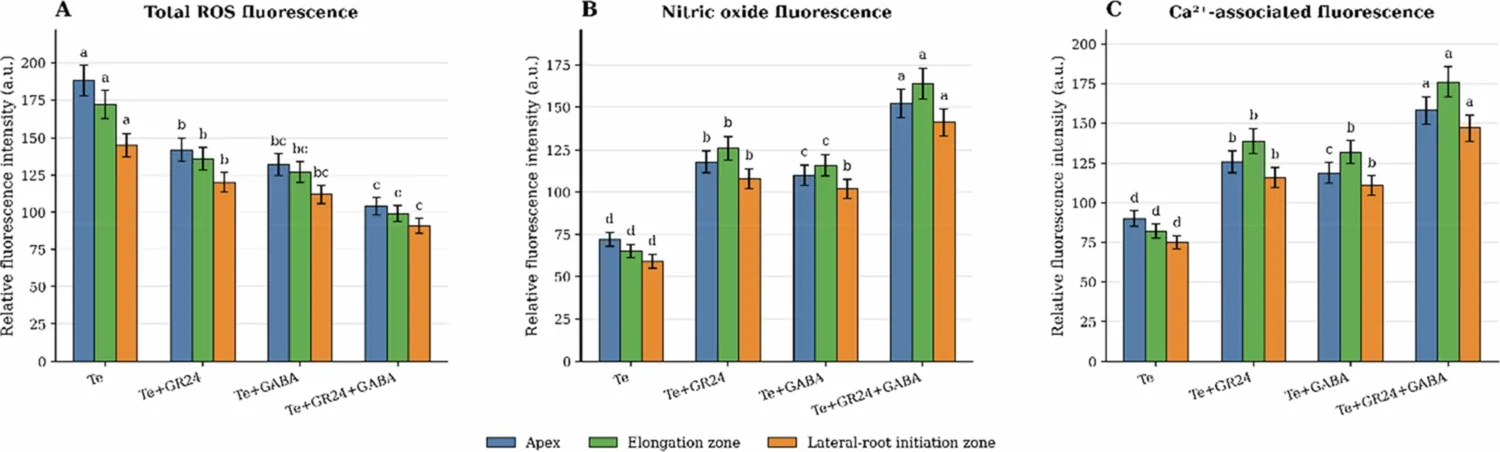
Spatial distribution of reactive oxygen species (ROS), nitric oxide (NO), and calcium (Ca^2^⁺)-associated signals in wheat root tips following foliar GR24 and GABA application under tellurite stress. (A) Total ROS detected with 2′,7′-dichlorodihydrofluorescein diacetate (H₂DCFDA); (B) NO detected with 4-amino-5-methylamino-2′,7′-difluorofluorescein diacetate (DAF-FM DA); and (C) Ca^2^⁺-associated fluorescence detected with a single-wavelength Ca^2^⁺-responsive probe. The treatments used were: C, untreated control; GR24, 1  µM GR24; GABA, 1  mM GABA; GR24 + GABA, combined treatment; Te, 60  mg tellurite kg^−1^ soil; and the corresponding Te + foliar-treatment combinations. Signals were quantified using identical imaging settings in the root apex, elongation zone, and lateral-root initiation zone. Six roots distributed across five independent pots were examined per treatment; scale bars = 200 µm.

Tellurite generated a rapid but transient oxidative burst (Figures 1 and 2). Root H₂O₂ increased from 3.10  µmol g^−1^ FW at 0 h to 4.72  µmol g^−1^ FW at 24 h, while superoxide production increased from 0.82 to 1.31 nmol g^−1^ FW min^−1^. The combined GR24 + GABA treatment limited the corresponding 24-h values to 2.94 and 0.79, representing reductions of 37.7% and 39.7%, respectively, relative to tellurite alone. This attenuation did not abolish signaling: at 24 h, NO increased from 0.36 under tellurite alone to 0.75 nmol equivalents g^−1^ FW under Te + GR24 + GABA, and Ca^2^⁺-associated fluorescence increased from 1.31 to 2.06 arbitrary units. Spatial imaging supported the biochemical measurements. The relative total-ROS fluorescence in the root apex declined from 188 under tellurite alone to 104 under the combined treatment, whereas NO- and Ca^2^⁺-associated fluorescence increased from 72 to 152 and from 90 to 158, respectively. Thus, GR24 and GABA shifted the early response from sustained ROS accumulation toward a coordinated ROS-NO-Ca^2^⁺ signature.

### 3.2. Combined application restores cellular redox and energy balance

**Figure 3.**
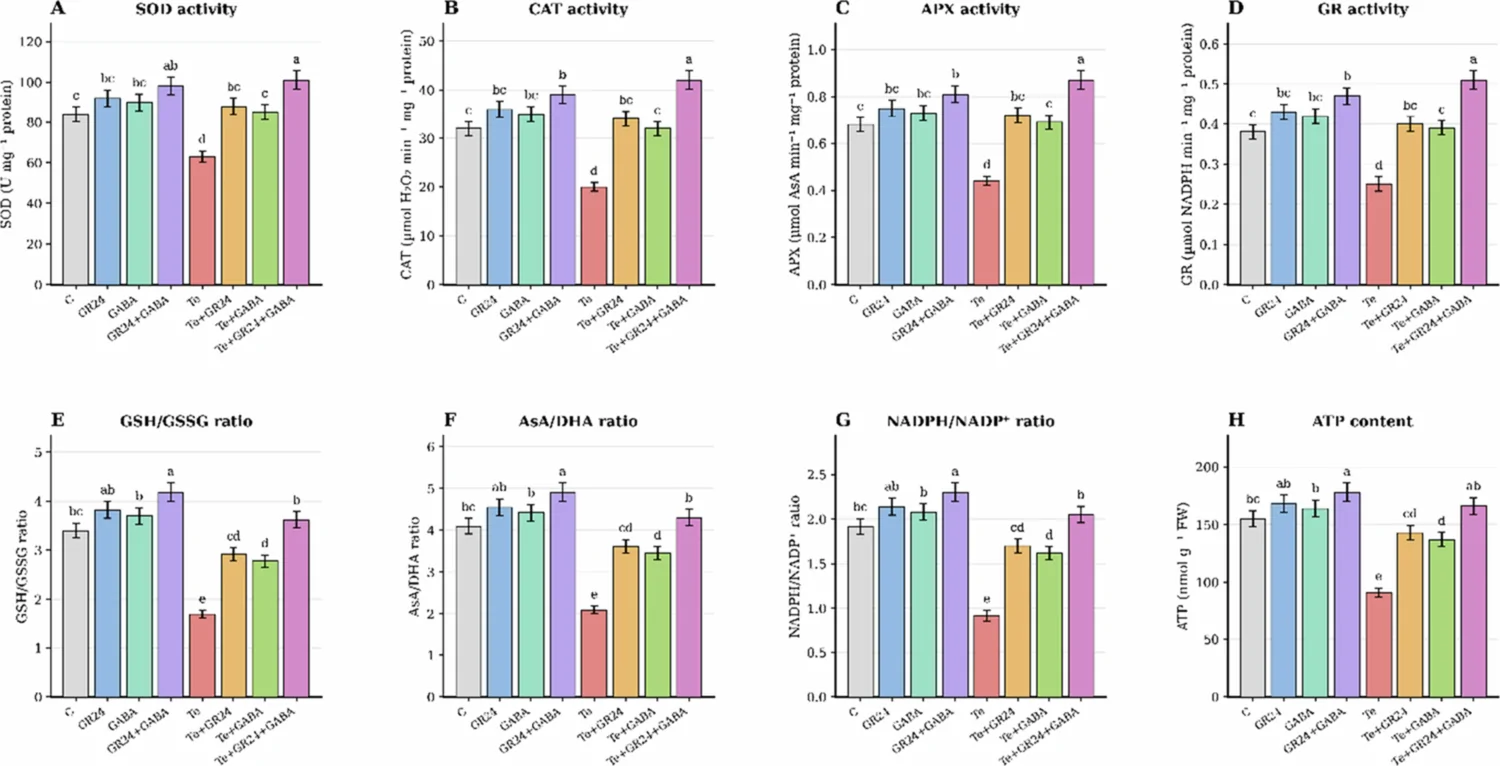
Effects of foliar GR24 and GABA on the antioxidant defense, cellular redox balance, and energy status of wheat under tellurite stress. (A) Superoxide dismutase (SOD); (B) catalase (CAT); (C) ascorbate peroxidase (APX); (D) glutathione reductase (GR) activity; (E) reduced-to-oxidized glutathione ratio (GSH/GSSG); (F) reduced ascorbate-to-dehydroascorbate ratio (AsA/DHA); (G) reduced-to-oxidized nicotinamide adenine dinucleotide phosphate ratio (NADPH/NADP⁺); and (H) adenosine triphosphate (ATP) content. The treatments used were: C, untreated control; GR24, 1 µM GR24; GABA, 1 mM GABA; GR24 + GABA, combined treatment; Te, 60 mg tellurite kg^−1^ soil; and the corresponding Te + foliar-treatment combinations. The values are the means ± standard deviation of five independent pots. Different lowercase letters indicate significant differences among treatments according to Tukey's honestly significant difference test at *p* < 0.05.

Tellurite suppressed the antioxidant capacity and depleted cellular reducing power. Relative to the control, SOD activity declined from 84 to 63 U mg^−1^ protein, CAT from 32 to 20  µmol H₂O₂ min^−1^ mg^−1^ protein, and the GSH/GSSG ratio decreased from 3.40 to 1.70. Foliar GR24 and GABA individually improved these variables, but their combined application produced the strongest recovery. In Te + GR24 + GABA plants, the SOD, CAT, APX, and GR activities reached 101, 42, 0.87, and 0.51 activity units, respectively. The GSH/GSSG, AsA/DHA, and NADPH/NADP⁺ ratios increased to 3.62, 4.31, and 2.05, compared with the values of 1.70, 2.10, and 0.92, respectively, under tellurite alone. Similarly, the ATP content increased from 91 to 166 nmol g^−1^ FW, an improvement of 82.4%. These changes indicate simultaneous recovery of antioxidant capacity, redox buffering, and energy availability (Figure 3).

### 3.3. GR24-GABA treatment limits oxidative and membrane damage

Tellurite caused pronounced lipid, protein, and membrane injury. Compared with the control, the MDA content increased from 18.42 to 42.63 nmol g^−1^ FW, the electrolyte leakage from 19.36% to 52.47%, and protein carbonyls from 3.18 to 7.82 nmol mg^−1^ protein, while the membrane stability index declined from 82.14% to 47.86%. GR24 and GABA each reduced this injury, with the combined treatment providing the greatest protection. Te + GR24 + GABA lowered MDA to 23.54 nmol g^−1^ FW, electrolyte leakage to 27.43%, and protein carbonyls to 4.13 nmol mg^−1^ protein, resulting in reductions of 44.8%, 47.7%, and 47.2%, respectively, relative to tellurite alone. Membrane stability simultaneously recovered to 75.28%, representing a 57.3% improvement over the tellurite-only treatment (Table 3).

**Table 3.** Oxidative, protein, and membrane damage in wheat following foliar GR24 and GABA application under tellurite stress.

Treatment	MDA (nmol g^−1^ FW)	Electrolyte leakage (%)	Protein carbonyls (nmol mg^−1^ protein)	Membrane stability index (%)
C	18.42 ± 1.31^d^	19.36 ± 1.48^d^	3.18 ± 0.24^d^	82.14 ± 3.26^a^
GR24	16.75 ± 1.16^d^	17.28 ± 1.35^d^	2.86 ± 0.21^d^	85.63 ± 2.91^a^
GABA	17.21 ± 1.24^d^	17.94 ± 1.42^d^	2.97 ± 0.23^d^	84.27 ± 3.04^a^
GR24+GABA	15.38 ± 1.09^d^	15.82 ± 1.21^d^	2.61 ± 0.19^d^	87.46 ± 2.78^a^
Te	42.63 ± 2.87^a^	52.47 ± 3.64^a^	7.82 ± 0.51^a^	47.86 ± 3.71^d^
Te+GR24	29.18 ± 2.06^b^	35.62 ± 2.48^b^	5.24 ± 0.38^b^	66.93 ± 3.42^c^
Te+GABA	31.46 ± 2.23^b^	38.15 ± 2.69^b^	5.61 ± 0.41^b^	63.78 ± 3.55^c^
Te+GR24+GABA	23.54 ± 1.72^c^	27.43 ± 1.96^c^	4.13 ± 0.31^c^	75.28 ± 3.18^b^

Values are means ± SD of five biological replicates. Superscript letters were determined using Tukey HSD at p < 0.05 (HSD = 3.71, 4.46, 0.67, and 6.65 for the four columns, respectively).

### 3.4. Treatments remodel root architecture and spatial exploration

Tellurite strongly restricted root-system development. The total root length declined from 542  cm plant^−1^ in the control to 306  cm plant^−1^ under tellurite, a reduction of 43.5%, while surface area and root volume declined from 116 to 67  cm^2^ plant^−1^ and from 5.80 to 3.30  cm^3^ plant^−1^, respectively. The combined foliar treatment restored the total root length to 536  cm plant^−1^, the surface area to 118  cm^2^ plant^−1^, and the volume to 5.90  cm^3^ plant^−1^. Relative to tellurite alone, these values represented increases of 75.2%, 76.1%, and 78.8%, respectively. Te + GR24 + GABA also increased lateral-root number from 42.4 to 86.8 plant^−1^, root tips from 139.6 to 276.2 plant^−1^, the maximum rooting depth from 19.4 to 32.7  cm, and the exploration index from 0.25 to 0.45  cm^2^ cm^−1^. The root-growth angle increased from 20.7° to 34.1°, indicating recovery of horizontal soil exploration as well as rooting depth (Table 4). In the absence of tellurite, GR24 alone increased lateral-root number from 84.2 to 96.6 plant^−1^, and the combination raised it to 104.8 plant^−1^.

**Table 4.** Root architectural, spatial-exploration, and behavioral traits of wheat under tellurite stress.

Treatment	Total root length (cm)	Surface area (cm^2^)	Root volume (cm^3^)	Mean diameter (mm)	Lateral roots	Root tips	Max depth (cm)	Growth angle (°)	Exploration index
C	542.0 ± 26.8^bc^	116.0 ± 6.2^b^	5.80 ± 0.31^b^	0.73 ± 0.04^ab^	84.2 ± 5.6^c^	268.4 ± 14.7^c^	31.8 ± 1.9^bc^	32.4 ± 2.1^bc^	0.42 ± 0.03^bcd^
GR24	594.0 ± 29.5^ab^	128.0 ± 6.8a^b^	6.40 ± 0.34^ab^	0.76 ± 0.04^ab^	96.6 ± 6.3^ab^	301.8 ± 16.5^ab^	35.2 ± 2.1^ab^	35.8 ± 2.3^ab^	0.48 ± 0.03^ab^
GABA	579.0 ± 28.7^abc^	124.0 ± 6.5^b^	6.20 ± 0.33^ab^	0.75 ± 0.04^ab^	92.8 ± 6.0^bc^	291.2 ± 15.9^bc^	34.1 ± 2.0^ab^	34.6 ± 2.2^ab^	0.46 ± 0.03^ab^
GR24 + GABA	628.0 ± 31.2^a^	137.0 ± 7.2^a^	6.80 ± 0.36^a^	0.79 ± 0.05^a^	104.8 ± 6.8^a^	326.6 ± 17.8^a^	37.6 ± 2.2^a^	38.2 ± 2.4^a^	0.52 ± 0.04^a^
Te	306.0 ± 17.4^e^	67.0 ± 4.1^d^	3.30 ± 0.21^d^	0.61 ± 0.04^c^	42.4 ± 3.7^e^	139.6 ± 9.8^e^	19.4 ± 1.4^e^	20.7 ± 1.7^e^	0.25 ± 0.02^e^
Te + GR24	474.0 ± 24.9^d^	103.0 ± 5.7^c^	5.10 ± 0.28^c^	0.70 ± 0.04^b^	72.6 ± 5.1^d^	232.4 ± 13.2^d^	28.6 ± 1.8^cd^	29.8 ± 2.0^cd^	0.39 ± 0.03^cd^
Te + GABA	448.0 ± 23.7^d^	96.0 ± 5.3^c^	4.80 ± 0.27^c^	0.68 ± 0.04^bc^	67.8 ± 4.8^d^	216.8 ± 12.5^d^	26.9 ± 1.7^d^	27.5 ± 1.9^d^	0.36 ± 0.03^d^
Te + GR24 + GABA	536.0 ± 27.5^c^	118.0 ± 6.3^b^	5.90 ± 0.32^b^	0.74 ± 0.04^ab^	86.8 ± 5.8^bc^	276.2 ± 15.1^bc^	32.7 ± 2.0^b^	34.1 ± 2.2^abc^	0.45 ± 0.03^bc^

Values are means ± SD of five biological replicates. Superscript letters were determined using Tukey HSD at *p* < 0.05. Root-growth angle was measured relative to the vertical axis; a larger angle indicates greater horizontal soil exploration.

### 3.5. GR24 and GABA improve root-hair and gravitropic responses

**Figure 4.**
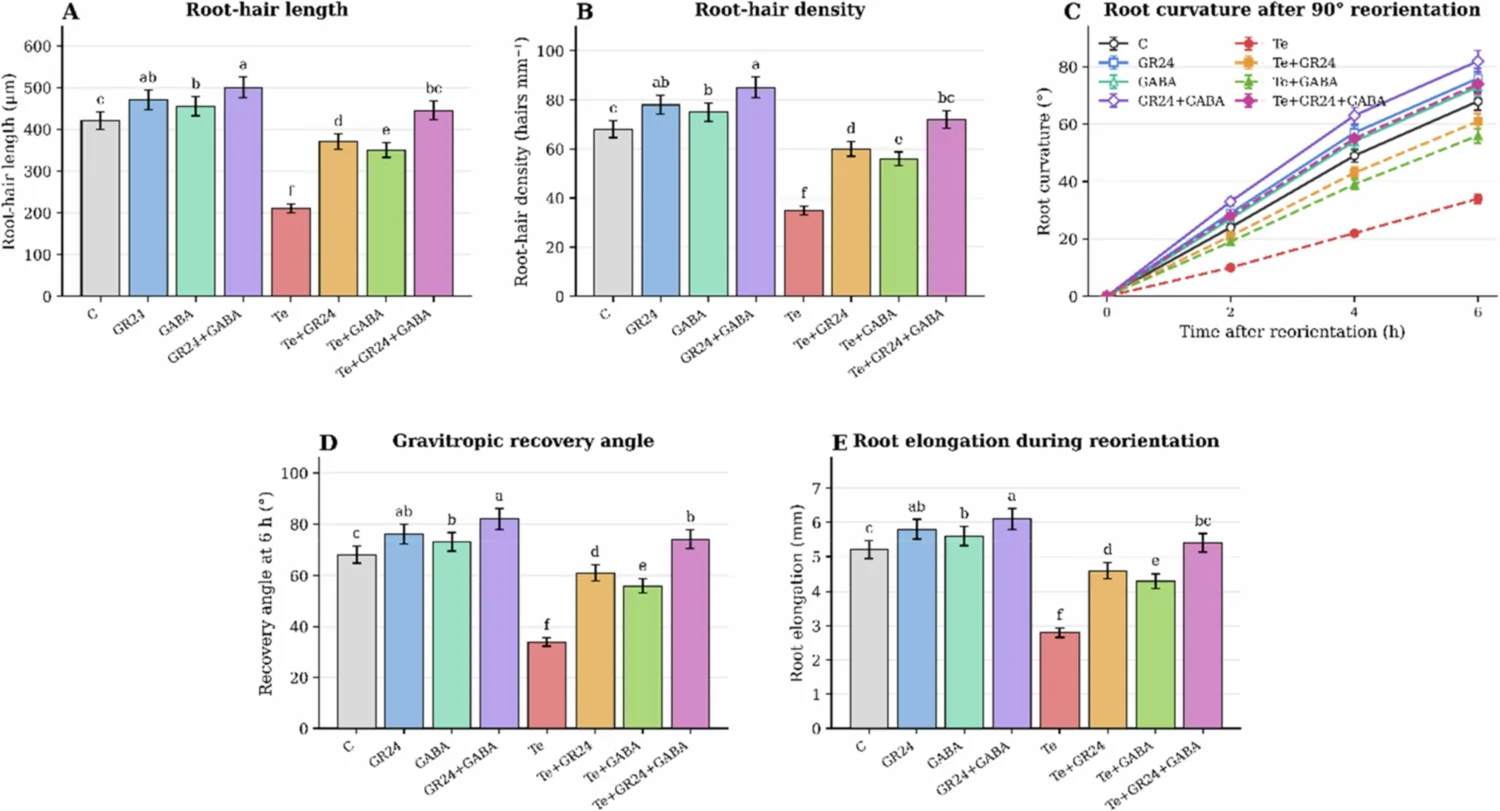
Root-hair development and gravitropic recovery of wheat seedlings following foliar GR24 and GABA application under tellurite stress. (A) Root-hair length; (B) root-hair density; (C) root curvature at 0, 2, 4, and 6 h after 90° reorientation; (D) gravitropic recovery angle at 6 h; and (E) root elongation during reorientation. The treatments used were: C, untreated control; GR24, 1  µM GR24; GABA, 1  mM GABA; GR24 + GABA, combined treatment; Te, 60  mg tellurite kg^−1^ soil; and the corresponding Te + foliar-treatment combinations. Six roots distributed across five independent pots were assessed per treatment. The values are the means ± standard deviation; in bar-chart panels, different lowercase letters indicate significant differences among the treatments according to Tukey's honestly significant difference test at *p* < 0.05.

Tellurite reduced root-hair length from 420 to 210 µm and root-hair density from 68 to 35 hairs mm^−1^. It also slowed gravitropic recovery: the final curvature at 6 h decreased from 68° in the control to 34°, and elongation during reorientation declined from 5.2 to 2.8 mm. Te + GR24 + GABA increased root-hair length to 445 µm and the density to 72 hairs mm^−1^, corresponding to improvements of 111.9% and 105.7% relative to tellurite alone. Root curvature reached 74° at 6 h, and the elongation reached 5.4 mm, increasing by 117.6% and 92.9%, respectively. GR24 alone generally produced a slightly stronger response than GABA alone, but the combined treatment most consistently restored both absorptive root-hair development and directional root behavior (Figure 4).

### 3.6. Membrane energization and proton extrusion are restored

**Figure 5.**
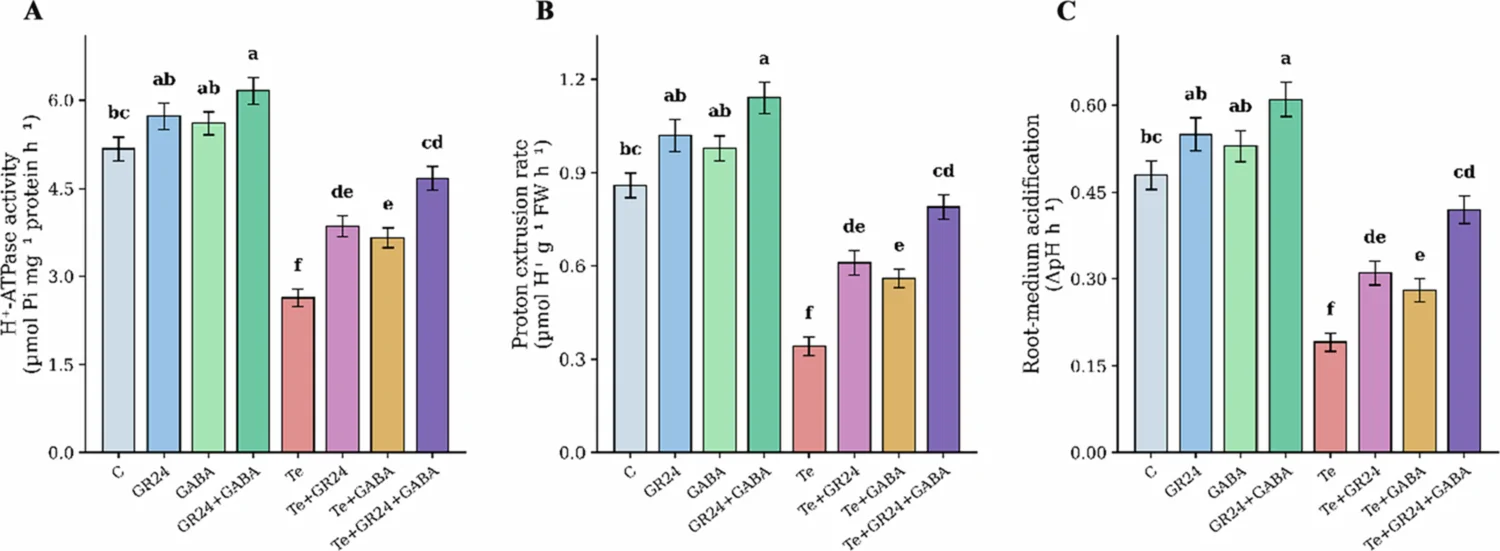
Effects of foliar GR24 and GABA on root membrane energization and proton transport in wheat under tellurite stress. (A) Plasma-membrane proton-translocating adenosine triphosphatase (H⁺-ATPase) activity; (B) proton-extrusion rate; and (C) root-medium acidification. The treatments used were: C, untreated control; GR24, 1  µM GR24; GABA, 1  mM GABA; GR24 + GABA, combined treatment; Te, 60  mg tellurite kg^−1^ soil; and the corresponding Te + foliar-treatment combinations. The values are the means ± standard deviation of five independent pots. Different lowercase letters indicate significant differences among treatments according to Tukey's honestly significant difference test at *p* < 0.05.

Tellurite reduced plasma-membrane H⁺-ATPase activity from 5.18 to 2.63  µmol Pi mg^−1^ protein h^−1^. The associated proton-extrusion rate declined from 0.86 to 0.34  µmol H⁺ g^−1^ FW h^−1^, and root-medium acidification declined from 0.48 to 0.19 ΔpH h^−1^. Te + GR24 + GABA restored these variables to 4.68, 0.79, and 0.42, respectively. Relative to tellurite alone, the combined treatment increased H⁺-ATPase activity by 78.0%, proton extrusion by 132.4%, and medium acidification by 121.1%. The close parallel among the three measurements indicates that biochemical activation of the proton pump translated into functional rhizosphere acidification (Figure 5).

### 3.7. Root exudation modifies rhizosphere chemistry and tellurium availability

**Figure 6.**
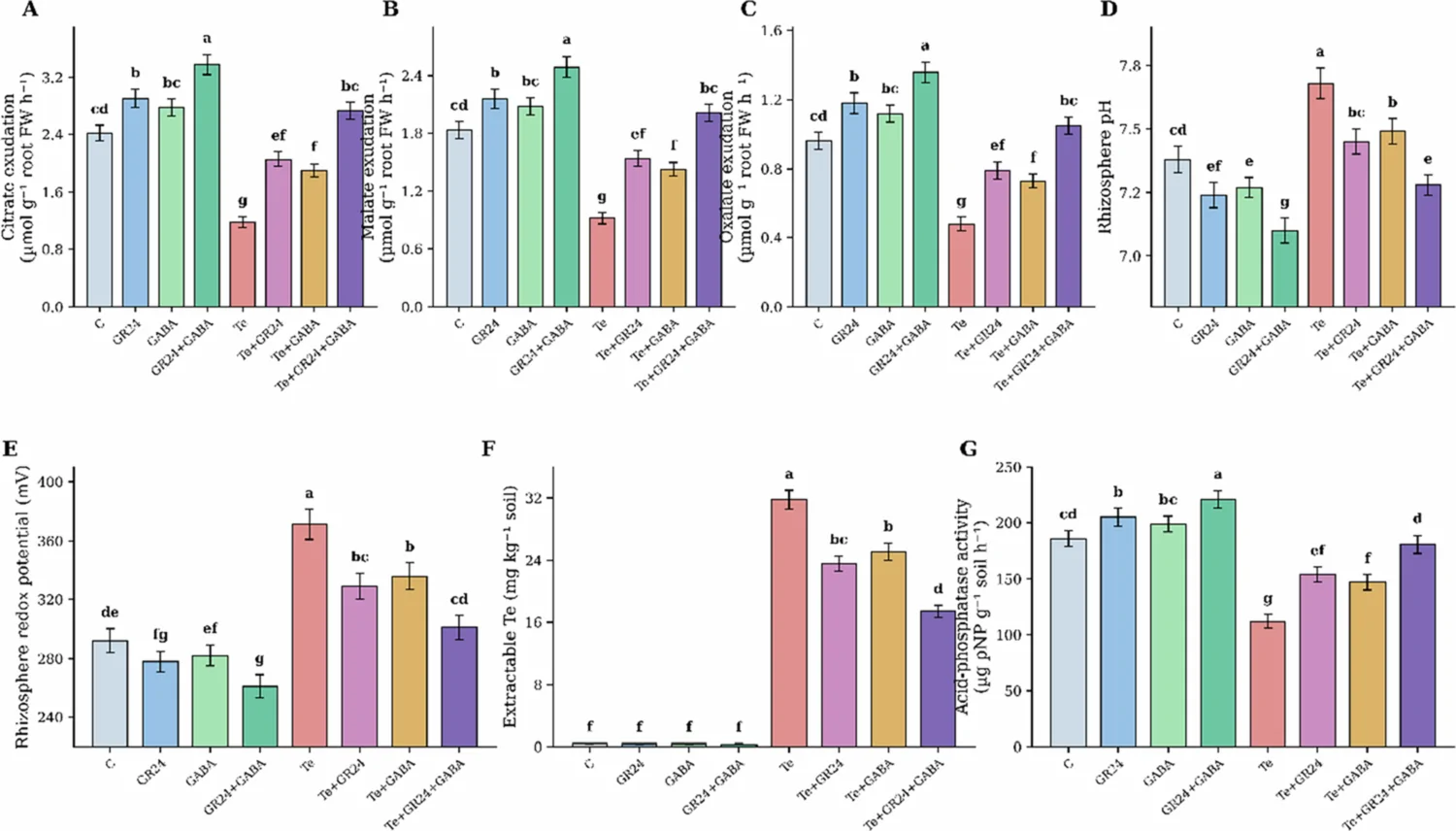
Root-exudate composition and rhizosphere properties of wheat following foliar GR24 and GABA application under tellurite stress. (A) Citrate; (B) malate; (C) oxalate exudation; (D) rhizosphere pH; (E) rhizosphere redox potential; (F) extractable tellurium (Te); and (G) acid-phosphatase activity. The treatments used were: C, untreated control; GR24, 1  µM GR24; GABA, 1  mM GABA; GR24 + GABA, combined treatment; Te, 60  mg tellurite kg^−1^ soil; and the corresponding Te + foliar-treatment combinations. Exudates were collected at final harvest, 30 d after the last foliar application, and expressed per unit root dry mass and collection time. The values are the means ± standard deviation of five independent pots. Different lowercase letters indicate significant differences among treatments according to Tukey's honestly significant difference test at *p* < 0.05.

Tellurite decreased citrate, malate, and oxalate exudation from 2.42, 1.83, and 0.96 to 1.18, 0.92, and 0.48  µmol g^−1^ root FW h^−1^, respectively. Te + GR24 + GABA increased their release to 2.73, 2.01, and 1.05  µmol g^−1^ root FW h^−1^. The enhanced organic-acid flux was accompanied by a decline in rhizosphere pH from 7.68 under tellurite alone to 7.28 and a decrease in redox potential from 371 to 301 mV. Te declined from 31.8 to 17.4  mg kg^−1^ soil, equivalent to a 45.3% reduction, whereas acid-phosphatase activity increased from 112 to 181  µg pNP g^−1^ soil h^−1^. GR24 and GABA alone produced intermediate responses. Exudation was measured at the final harvest, 30 d after the last foliar application, and therefore reflects a sustained physiological state rather than an acute response to the applied compounds (Figure 6).

### 3.8. Mineral selectivity is improved under tellurite stress

Tellurite reduced root S, P, K, Ca, Fe, and Zn from 3.42, 2.61, 18.40, and 7.25 g kg^−1^ DW, 168.0 and 42.3 mg kg^−1^ DW in the control to 2.05, 1.54, 10.80, and 4.62 g kg^−1^ DW, 112.0 and 29.5 mg kg^−1^ DW, respectively. Te + GR24 + GABA restored these concentrations to 3.25, 2.55, 17.60, and 7.02 g kg^−1^ DW, 171.0, and 43.5  mg kg^−1^ DW, respectively. The combined treatment also increased the molar S/Te, P/Te, K/Te, Ca/Te, Fe/Te and Zn/Te ratios from 211.4, 164.3, 913.0, 381.2, 6.63, and 1.49 under tellurite alone to 260.8, 211.8, 1,157.0, 450.6, 7.88, and 1.71, respectively, corresponding to improvements of 23.4%, 28.9%, 26.7%, 18.2%, 18.9%, and 14.8%. The improvement was broad rather than element-specific and was largest for P/Te and K/Te rather than for S/Te. These changes show that GR24 + GABA improved nutrient acquisition relative to Te accumulation rather than merely increasing total elemental uptake (Tables 5 and 6).

**Table 5.** Mineral concentrations in root and shoot tissues of wheat.

Treatment	Root S (g kg^−1^)	Shoot S (g kg^−1^)	Root *P* (g kg^−1^)	Root K (g kg^−1^)	Root Ca (g kg^−1^)	Root Fe (mg kg^−1^)	Root Zn (mg kg^−1^)
C	3.42 ± 0.15 ^bc^	3.78 ± 0.17^bc^	2.61 ± 0.12bc	18.40 ± 0.72^bc^	7.25 ± 0.31^bc^	168.0 ± 7.2^b^	42.3 ± 1.8^b^
GR24	3.65 ± 0.16^ab^	4.02 ± 0.18^ab^	2.79 ± 0.13ab	19.60 ± 0.81^ab^	7.68 ± 0.34^ab^	178.0 ± 7.8^ab^	45.1 ± 1.9^ab^
GABA	3.57 ± 0.15^ab^	3.94 ± 0.16^ab^	2.73 ± 0.11abc	19.10 ± 0.76^ab^	7.51 ± 0.32^abc^	174.0 ± 7.1^ab^	44.0 ± 1.7^ab^
GR24 + GABA	3.78 ± 0.17^a^	4.16 ± 0.19^a^	2.88 ± 0.13^a^	20.20 ± 0.84^a^	7.92 ± 0.35^a^	184.0 ± 8.0^a^	46.7 ± 2.0^a^
Te	2.05 ± 0.10^e^	2.16 ± 0.11^e^	1.54 ± 0.08^e^	10.80 ± 0.49^e^	4.62 ± 0.22^e^	112.0 ± 5.6^d^	29.5 ± 1.4^d^
Te + GR24	2.72 ± 0.12^d^	2.96 ± 0.13^d^	2.03 ± 0.09^d^	14.10 ± 0.61^d^	5.68 ± 0.26^d^	139.0 ± 6.3^c^	35.8 ± 1.6^c^
Te + GABA	2.61 ± 0.11^d^	2.84 ± 0.12^d^	1.94 ± 0.09^d^	13.60 ± 0.58^d^	5.42 ± 0.25^d^	134.0 ± 6.1^c^	34.4 ± 1.5^c^
Te + GR24 + GABA	3.25 ± 0.14^c^	3.55 ± 0.15^c^	2.55 ± 0.11^c^	17.60 ± 0.69^c^	7.02 ± 0.30^c^	171.0 ± 7.4^ab^	43.5 ± 1.8^ab^

Values are means ± SD of five biological replicates. Letters were determined using Tukey HSD at *p* < 0.05.

**Table 6.** Mineral-to-tellurium selectivity ratios in wheat roots, calculated on a molar basis. Ratios were not calculated for non-tellurite treatments because tissue Te was below the reliable quantification limit. Percentage improvement relative to the tellurite-only treatment is given in the final row.

Treatment	S/Te	P/Te	K/Te	Ca/Te	Fe/Te	Zn/Te
Te	211.4^d^	164.3^d^	913.0^d^	381.2^d^	6.63^c^	1.49^c^
Te + GR24	244.9^b^	189.2^b^	1,041.0^b^	409.1^bc^	7.19^b^	1.58^b^
Te + GABA	242.7^bc^	186.8^bc^	1,037.0^bc^	403.2^c^	7.15^b^	1.57^b^
Te + GR24 + GABA	260.8^a^	211.8^a^	1,157.0^a^	450.6^a^	7.88^a^	1.71^a^
**Improvement (%)**	+23.4	+28.9	+26.7	+18.2	+18.9	+14.8

Different lowercase letters (a, b, c, and d) within the same column indicate significant differences among treatments according to Tukey’s HSD test at *p* < 0.05.

### 3.9. Root sequestration restricts tellurium movement to shoots

Tellurite-only plants accumulated 38.6  mg Te kg^−1^ DW in roots and 15.8  mg Te kg^−1^ DW in shoots, resulting in a root-to-shoot translocation factor of 0.409. The combined GR24 + GABA treatment increased root Te accumulation to 49.6  mg kg^−1^ DW but reduced the shoot Te concentration to 6.1  mg kg^−1^ DW. Consequently, the translocation factor declined to 0.123, while Te exclusion reached 61.4%. Subcellular partitioning further explained this restriction in Te movement. The proportion of root Te retained in the cell-wall fraction increased from 42% under tellurite alone to 65% under the combined treatment. In contrast, the organelle and soluble fractions declined from 30% and 28% to 18% and 17%, respectively. Individual GR24 and GABA applications produced intermediate responses. Therefore, the reduction in shoot Te was associated with greater root retention and preferential redistribution of Te toward the cell-wall fraction rather than reduced root uptake (Table 7).

**Table 7.** Tellurium accumulation, subcellular partitioning, translocation, and exclusion in wheat following foliar GR24 and GABA application under tellurite stress.

Treatment	Root Te (mg kg^−1^ DW)	Shoot Te (mg kg^−1^ DW)	Translocation factor	Te exclusion (%)	Cell-wall fraction (% of root Te)	Organelle fraction (% of root Te)	Soluble fraction (% of root Te)
Te	38.6 ± 1.7 c	15.8 ± 0.72 a	0.409 ± 0.021 a	–	42	30	28
Te + GR24	44.2 ± 1.9 b	9.8 ± 0.46 b	0.222 ± 0.014 b	38.0 ± 2.1 b	54	24	22
Te + GABA	42.8 ± 1.8 b	10.6 ± 0.49 b	0.248 ± 0.015 b	32.9 ± 1.9 c	51	25	24
Te + GR24 + GABA	49.6 ± 2.1 a	6.1 ± 0.31 c	0.123 ± 0.009 c	61.4 ± 2.7 a	65	18	17

Values are means ± SD of five independent biological replicates. Different superscript letters within a column indicate significant differences according to Tukey’s HSD test at *p* < 0.05.

The em dash indicates that Te exclusion was not applicable to the Te-only reference treatment. Te exclusion was calculated relative to this treatment. Subcellular fractions are expressed as percentages of total root Te and sum to 100% within each treatment.

### 3.10. Integrated analyses connect early signals with root and mineral responses

**Figure 7.**
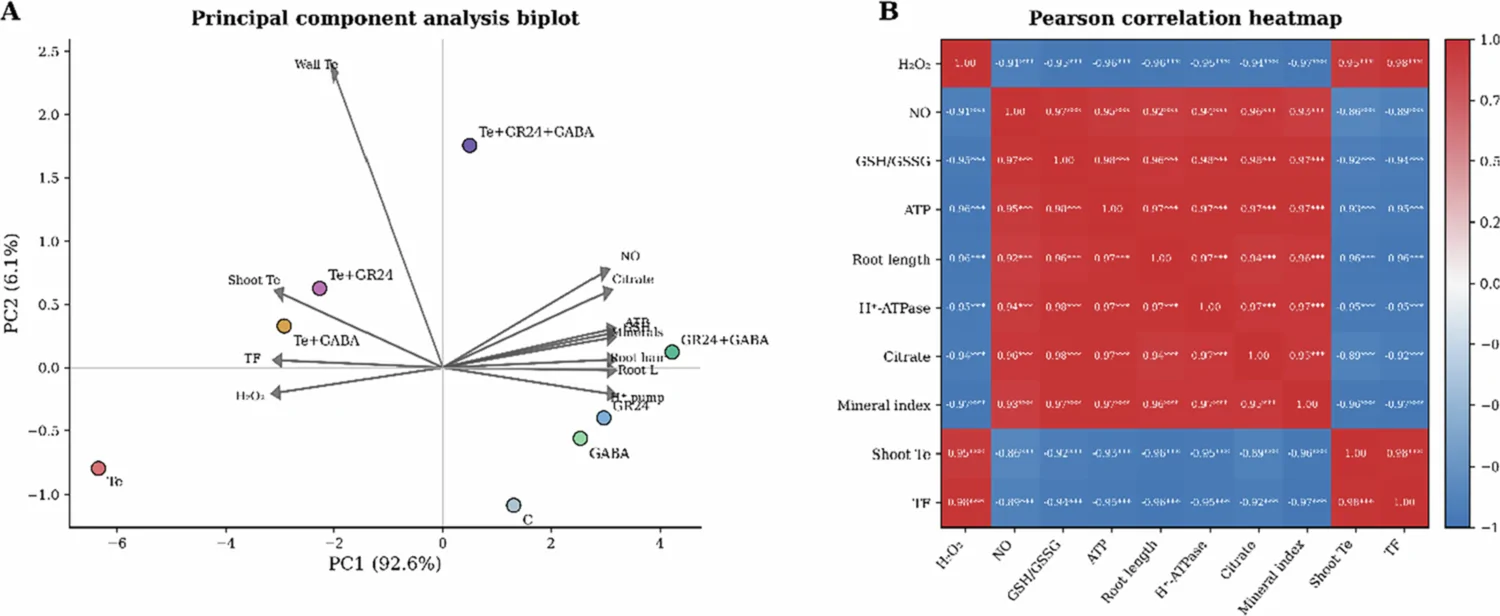
Integrated relationships among early signaling, redox regulation, root behavior, mineral homeostasis, and tellurium partitioning across eight treatment combinations. (A) Principal component analysis (PCA) biplot, in which treatment positions show multivariate similarity and vector direction and length represent trait loadings; and (B) Pearson correlation heatmap, in which color and magnitude indicate the direction and strength of pairwise associations. The treatments were: C, untreated control; GR24, 1  µM GR24; GABA, 1  mM GABA; GR24 + GABA, combined treatment; Te, 60  mg tellurite kg^−1^ soil; and the corresponding Te + foliar-treatment combinations. Both analyses used standardized treatment means (*n* = 8 treatments), not pot-level replicates; therefore, associations should be interpreted as treatment-level patterns. *p < 0.05, **p < 0.01 and ***p < 0.001.

Principal component analysis separated the tellurite-only treatment from the unstressed and GR24/GABA-treated groups, with PC1 and PC2 explaining 92.6% and 6.1% of the total variation, respectively. H₂O₂, shoot Te, and the translocation factor are loaded in the opposite direction to NO, GSH/GSSG, ATP, H⁺-ATPase activity, root length, citrate exudation, and the mineral index. Pearson analysis supported these associations: H₂O₂ was negatively correlated with root length (r = −0.96), H⁺-ATPase activity (r = −0.95), and mineral status (r = −0.97), whereas shoot Te was positively correlated with H₂O₂ (r = 0.95) and the translocation factor (r = 0.98). Te + GR24 + GABA shifted toward the beneficial redox, root, and mineral vectors while remaining associated with cell-wall Te retention. Because both the ordination and the correlation analysis were conducted on standardized treatment means (*n* = 8), the proportion of variance explained and the magnitude of the correlation coefficients are higher than would be obtained from pot-level data and should not be interpreted as replicate-level associations. The multivariate pattern, therefore, connected early signal regulation with later membrane, root and elemental outcomes (Figure 7).

## 4. Discussion

### 4.1. Tellurite triggers an early signaling response distinct from later oxidative injury

The time-resolved design distinguishes an early, regulated signaling burst from the oxidative injury evident at harvest, two processes that a single endpoint measurement would conflate.^13^ ^,^ ^17^ Tellurite chemistry makes this distinction important because the oxyanion can oxidize thiols and generate superoxide during intracellular reduction.^6^ ^,^ ^8^ The concurrent activation of NADPH oxidase and localization of ROS in the root apex therefore suggest that chemically driven oxidative pressure was accompanied by an active plant signaling response. Nevertheless, the present data distinguish temporal association, not the precise enzymatic contribution to ROS production.^16^ ^,^ ^23^

Nitric oxide and calcium complete this picture. Early NO transients under metalloid stress have been described in several species, and NO interacts with the glutathione pool through S-nitrosoglutathione, which links the NO signal directly to the thiol system that tellurite depletes.^18^ ^,^ ^20^ ^,^ ^75^ The Ca^2^⁺-associated fluorescence detected in the apical region, which rose from 1.31 to 2.06 arbitrary units under the combined treatment, is consistent with the established feedback between cytosolic Ca^2^⁺ elevation and NADPH oxidase activation.^21^ ^,^ ^22^ It should be stated plainly, however, that without a calibrated ratiometric indicator or the use of channel inhibitors, these data describe an association and not a demonstrated causal sequence.

Placing tellurite within the broader biology of toxic-element stress reveals both shared and distinctive responses. Cadmium, arsenic, and chromium commonly inhibit root elongation, disturb photosynthesis and mineral nutrition, increase membrane leakage and lipid peroxidation, and activate Ca^2^⁺, ROS, nitric oxide, and antioxidant responses.^13^ ^,^ ^17^ ^,^ ^44^ ^,^ ^75^ The tellurite-induced rise in ROS and NO, depletion of reduced glutathione, loss of membrane function, and inhibition of root development resemble the responses reported for these better-studied toxic elements. The important difference is that similar physiological endpoints can arise from different entry and reaction chemistries. Cd^2^⁺ is a non-redox-active heavy-metal ion that enters through transport systems for essential divalent cations and promotes ROS mainly indirectly by displacing nutrients, binding protein sulfhydryl groups, and disrupting antioxidant and electron-transport processes. Tellurite is a redox-active oxyanion that can oxidize thiols directly and generate superoxide during intracellular reduction.^6-8^ Thus, the pronounced dependence of tellurite injury on the GSH/GSSG couple is chemically more immediate than the secondary redox imbalance generally produced by Cd.

Tellurite is more closely analogous to arsenate and chromate at the uptake level because all three occur as nutrient-like oxyanions. Arsenate exploits high-affinity phosphate transport; mutation of Arabidopsis PHT1;1 alters arsenate accumulation, and arsenate exposure induces oxidative-stress genes while disrupting phosphate-starvation signaling.^76^ ^,^ ^77^ Chromate similarly competes with sulfate, and genetic evidence identifies the high-affinity sulfate transporter SULTR1;2 as a major route for Cr(VI) uptake in Arabidopsis.^78^ These established examples show how toxic oxyanions combine nutrient mimicry with oxidative damage. The present tellurite results also showed impaired S and P statuses, but the comparable recovery of S/Te, P/Te, K/Te, and other mineral/Te ratios does not identify a single transporter. Tellurite, therefore, shares the nutrient-competition, ROS production, thiol depletion, and root-dominant injury typical of metalloid and Cr(VI) stress, while remaining distinct because its uptake routes in higher plants, regulatory thresholds, field exposure ranges, and long-distance transport are far less resolved. This comparison supports interpreting GR24 + GABA-mediated recovery as a broad restoration of redox buffering, membrane energization, and root function rather than evidence for selective blockade of a specific tellurite transporter.

### 4.2. GR24 and GABA regulate the intensity and duration of the redox signal

The treatment effect is best interpreted from signal dynamics rather than from ROS suppression alone. GR24 + GABA preserved the early NO- and Ca^2^⁺-associated responses while accelerating the decline of excess H₂O₂ and superoxide, a pattern consistent with improved signal resolution rather than indiscriminate inhibition of the oxidative burst.^13^ ^,^ ^17^ The combined treatment may therefore allow stress perception and defense activation while limiting prolonged oxidative exposure.

The two compounds are unlikely to act identically. Strigolactone signaling has been linked to antioxidant capacity and to the modulation of ROS under several abiotic stresses, although the extent to which this reflects a direct action rather than a consequence of improved growth remains disputed, and the present data cannot settle that question.^27^ ^,^ ^79-82^ The closest published comparison is instructive: foliar GR24 at the same 1  µM concentration used here reduced cadmium uptake in barley, enhanced nitric oxide signaling, and activated the ascorbate–glutathione cycle,^36^ a combination that matches the present results closely in a related cereal. In contrast, GABA is positioned close to both the antioxidant system and primary metabolism: the GABA shunt supplies succinate to the tricarboxylic acid cycle when its oxidative branch is inhibited, which is precisely the condition expected when thiol-dependent enzymes are compromised by tellurite.^29^ ^,^ ^83^ NADPH/NADP⁺ reached 2.05 and ATP 166 nmol g^−1^ FW under the combined treatment, against 0.92 and 91 under tellurite alone, which is consistent with the metabolic support of the reductant supply on which the antioxidant system depends.^29^ ^,^ ^83^

The selection of GR24 and GABA was objectives-driven. GR24 is a synthetic strigolactone analog and an established experimental probe of D14-MAX2-SMXL signaling, a hormonal module that connects nutrient status and auxin distribution with root-system architecture and ROS-dependent stress acclimation.^24-28^ GABA was selected not merely as an antioxidant treatment, but also as a neurotransmitter-like plant signal and metabolic intermediate that can link stress-evoked Ca^2^⁺-ROS-NO dynamics with ALMT-family anion-channel regulation and a GABA-shunt-derived energy and reductant supply.^29^ ^,^ ^30^ ^,^ ^83^ Pairing GR24 with GABA, therefore, allowed us to test the interaction between a hormone-governed developmental pathway and a rapid neurotransmitter-like/metabolic pathway. This combination is particularly relevant to tellurite because the stress simultaneously disrupts thiol redox buffering, membrane energization, anion transport, and root development: GR24 was expected to favor developmental reprogramming and stress‒signal coordination, whereas GABA could reinforce redox metabolism, the cellular energy status, and membrane transport. Their co-application was consequently chosen to determine whether regulation at these complementary control points produces a broader protective response than either compound alone, rather than on the assumption that both treatments would simply increase antioxidant activity. The greater recovery under GR24 + GABA across signal resolution, root plasticity, plasma-membrane H⁺-ATPase activity, mineral/Te selectivity, and Te partitioning supports this rationale; however, direct molecular crosstalk between the strigolactone and GABA pathways remains to be verified experimentally.

A pathway-based interpretation can connect these responses without implying that the unmeasured molecular steps were demonstrated. GR24 is perceived through the canonical Dwarf14-More Axillary Growth2 (D14-MAX2) strigolactone signaling module, which promotes degradation of Suppressor of Max2-Like (SMXL) repressors and thereby modifies transcriptional programs governing auxin distribution, root branching, and nutrient-responsive root development.^24-28^ Under tellurite stress, this pathway could account for the recovery of lateral-root formation, root-hair development, and gravitropic reorientation. In parallel, stress-induced Ca^2^⁺ elevations can be decoded by Ca^2^⁺-dependent kinases that activate respiratory-burst oxidase homologs, generating the regulated ROS pulse required for acclimation.^21^ ^,^ ^22^ The observed increase in NADPH oxidase activity, followed by a decline in excess H₂O₂ and superoxide under GR24 + GABA, is therefore consistent with preservation of an early Ca^2^⁺-respiratory burst oxidase homologue (RBOH) signaling event followed by more efficient ROS removal rather than indiscriminate suppression of ROS production.

GABA provides a complementary metabolic and transport-related route. GABA signaling directly regulates plant aluminum-activated malate transporter (ALMT)-family anion channels, while the GABA shunt supplies succinate to the tricarboxylic-acid cycle and can help maintain NADPH and ATP generation when oxidative metabolism is disturbed.^29^ ^,^ ^30^ ^,^ ^83^ The higher NADPH/NADP⁺ and ATP status under GR24 + GABA would support glutathione reductase and the ascorbate‒glutathione cycle, facilitating the conversion of GSSG to GSH and the detoxification of excess H₂O₂.^61^ ^,^ ^84^ ^,^ ^85^ Nitric oxide may reinforce this redox adjustment through reversible S-nitrosylation of antioxidant and signaling proteins and through interaction with the glutathione/S-nitrosoglutathione (GSNO) pool.^18-20^ These established pathways provide a physiologically coherent explanation for the simultaneous preservation of NO and Ca^2^⁺-associated signals, recovery of the GSH/GSSG and AsA/DHA ratios, and reduction of terminal oxidative damage. Nevertheless, because D14-MAX2-SMXL components, ALMT activity, protein S-nitrosylation, and pathway-specific fluxes were not measured, these links are presented as a testable mechanistic framework rather than a proven causal chain.

The additivity or otherwise of the combined treatment is therefore diagnostic. The combination exceeded either compound alone for every architectural and redox variable measured, which makes complementary rather than overlapping modes of action the more parsimonious explanation, although this inference requires the tellurite × foliar-treatment interaction term to be significant, and that test must be reported explicitly. These findings are consistent with, but do not demonstrate, direct crosstalk between the two pathways, establishing that would require genetic or pharmacological intervention.

### 4.3. The ascorbate-glutathione balance underlies redox recovery

Redox ratios are more informative than total pools because the GSH/GSSG and AsA/DHA couples determine the cellular reducing capacity. Tellurite directly consumes reduced glutathione, making this pool both a defense and a target.^7^ ^,^ ^8^ ^,^ ^61^ ^,^ ^84^ ^,^ ^85^ The comparable recovery of both ratios under GR24 + GABA indicates the restoration of the ascorbate‒glutathione cycle as an integrated system rather than a selective effect on glutathione alone. This interpretation is also consistent with the enhanced glutathione reductase activity and maintenance of the NADPH supply required for GSH regeneration.

### 4.4. Root architectural plasticity as an adaptive behavioral response

The root traits measured here fall into two categories that should not be conflated. Total root length and biomass are largely a function of how much growth was possible; lateral root density, specific root length, growth angle, and the exploration index describe how the available growth was *allocated.* ^24^ A plant that produced fewer roots but distributed them differently has made a behavioral adjustment, and it is this distinction that justifies the framing of root plasticity as behavior rather than as a growth response. One aspect of the architectural response requires an explicit comment because it runs counter to the published direction of strigolactone action. Under phosphate-sufficient conditions, applied GR24 has repeatedly been shown to suppress lateral root primordium development and reduce lateral root density in Arabidopsis, an inhibition that weakens only under phosphate limitation^25^, and comparable suppression has been reported in Medicago truncatula and in rice secondary lateral roots.^26^ In the present experiment, conducted in fertilized soil characterized by available phosphorus, GR24 alone increased the lateral root number from 84.2 to 96.6 per plant, and the combined treatment raised it further. Three differences from the published systems may account for this. First, wheat is a cereal with a seminal and nodal root system whose developmental control differs from that of a taproot dicot. Second, the compound was applied to the foliage rather than supplied to the root medium, so the concentration reaching the root apex is unknown and is certainly lower than that in the agar-based systems in which suppression was characterized. Third, 1 µM lies at the low end of the effective range, and dose-dependent reversal of the GR24 effect on root development is well documented.^25^ The present design cannot discriminate among these possibilities, and the result should be reported as a species and delivery-specific observation rather than assimilated into the general strigolactone literature.

Gravitropic recovery provides evidence for a behavioral response that cannot be explained solely by greater root size, because reorientation requires perception, auxin redistribution, and differential elongation. The involvement of GR24 is mechanistically plausible through strigolactone‒auxin crosstalk, although it has not been tested directly.^25^ ^,^ ^26^ The parallel recovery of root-hair length and density expands the absorptive interface and offers a physical explanation for improved mineral acquisition that complements, but does not prove transporter-level regulation.

### 4.5. Membrane energization links root behavior to nutrient acquisition

The plasma-membrane H⁺-ATPase generates the proton‒motive force on which most secondary nutrient transport depends, and its inhibition therefore reduces the uptake of many nutrients simultaneously.^86^ ^,^ ^87^ Under tellurite stress, membrane depolarization and reduced proton extrusion offer a single mechanistic explanation for the broad decline in mineral concentrations, and the restoration of pump activity offers a correspondingly economical explanation for their broad recovery.^88^ The selectivity ratios address a question that concentrations alone cannot: a rise in the mineral/Te ratio demonstrates something that a rise in tissue mineral content does not, namely, that the plant discriminated more effectively between the nutrient and the toxic oxyanion.

Mechanistically, the recovery of plasma-membrane H⁺-ATPase activity provides the most direct bridge between redox protection and nutrient acquisition. The H⁺-ATPase establishes the membrane potential and proton gradient that energize H⁺-coupled nitrate, phosphate, and sulfate transporters and supports K⁺ and other cation uptake through voltage-dependent transport systems.^86^ ^,^ ^87^ The limitation of lipid peroxidation and maintenance of the ATP supply would be expected to preserve this pump, whereas enhanced proton extrusion would increase the driving force for nutrient uptake and influence rhizosphere pH. The concurrent recovery of H⁺-ATPase activity, proton extrusion, mineral concentrations, and mineral/Te selectivity is consistent with this mechanism. GABA-dependent regulation of ALMT channels may additionally alter organic-anion flux and rhizosphere chemistry,^30^ ^,^ ^87^ ^,^ ^88^ while GR24-associated remodeling of root hairs and lateral roots increases the absorbing surface on which these transport processes operate. This model explains why improvements occurred across several nutrients rather than through a single Te-specific transporter; however, transporter abundance and ion fluxes must be quantified before individual uptake systems can be assigned causal roles.

The broadly improved mineral/Te selectivity profile does not identify a single route of tellurite entry. Because recovery was not specific to sulfur, the pattern is more consistent with the general restoration of proton-motive-force-dependent nutrient acquisition together with independent restriction of Te in the rhizosphere and root cell wall. Evidence for sulfate-mediated tellurite handling is strongest in yeast,^10^ whereas equivalent transport evidence in higher plants is lacking. Competitive uptake assays and sulfate- or phosphate-transporter mutants are therefore needed to resolve the entry pathway.

### 4.6. Root exudates and cell-wall sequestration restrict tellurium mobility

Two complementary processes may restrict Te movement to the shoot. Increased organic-acid exudation and rhizosphere acidification could reduce the extractable Te pool through complexation or immobilization.^89^ However, GABA is known to inhibit the wheat malate channel TaALMT1, so the observed long-term malate response cannot be assigned directly to acute GABA regulation.^30^ ^,^ ^90^ ^,^ ^91^ The harvest-time measurement, foliar delivery, and contribution of citrate and oxalate may explain part of this discrepancy, but direct measurements of root GABA and malate flux, ideally using TaALMT1 near-isogenic lines, are required.

Internally, greater binding of Te to cell-wall carboxyl and hydroxyl groups would sequester the toxicant outside the protoplast.^92^ ^,^ ^93^ The combined pattern of greater root retention, reduced shoot accumulation, lower translocation, and redistribution away from organelle and soluble fractions therefore indicates effective sequestration rather than unrestricted accumulation. Subcellular partitioning is essential to this interpretation because total root Te alone cannot distinguish detoxifying retention from increased cellular exposure.

### 4.7. An integrated physiological model

Taken together, the results support a sequence in which tellurite entry through anion transport routes provokes an early, spatially localized ROS-NO signal in the root apex; foliar GR24 and GABA shorten and dampen the oxidative arm of this signal while preserving the NO and Ca^2^⁺ arms; improved glutathione and ascorbate regeneration, supported by maintained NADPH and ATP supply, restores redox potential; restored plasma-membrane H⁺-ATPase activity re-establishes the proton-motive force required for nutrient acquisition across the full range of transported elements; and altered exudation together with cell-wall binding restricts tellurium movement to the shoot. Root architectural and gravitropic recovery both depend on and reinforce this sequence. This model is supported by association and by physiological coherence, not by direct molecular evidence. In the absence of mutants, gene-expression data, or pharmacological inhibitors, the sequence is offered as a hypothesis consistent with the data rather than as a demonstrated pathway.

Viewed as an integrated physiological feedback model, the combined GR24 + GABA treatment appears to interrupt the self-reinforcing cycle of tellurite injury at several connected points. Strengthening of the ascorbate‒glutathione redox network and maintenance of NADPH and ATP availability would favor ROS detoxification, thereby limiting lipid peroxidation, protein oxidation, and electrolyte leakage. Better preservation of membrane integrity and the cellular energy status would, in turn, sustain plasma-membrane H⁺-ATPase activity and the proton-motive force needed for nutrient uptake. The resulting recovery of P, K, Ca, Fe, Zn, and S homeostasis can support antioxidant enzyme function, osmotic regulation, photosynthetic metabolism, and meristematic growth, linking redox protection directly with the restoration of plant nutritional status.

Root plasticity is both an outcome and a reinforcing component of this response. The recovery of total and lateral root growth, root-hair development, and gravitropic reorientation expands the soil volume explored, and the absorptive surface available for water and mineral acquisition, which can further sustain energy metabolism and antioxidant turnover. At the same time, modified rhizosphere chemistry and greater retention of Te in the root cell wall reduce the soluble Te pool and its movement to metabolically active compartments and shoots, lowering the continuing oxidative burden. Thus, antioxidant defense, nutrient homeostasis, and root-system plasticity should not be interpreted as independent endpoints; together, they form a mutually reinforcing physiological network in which improved redox control preserves membrane transport, restores nutrient acquisition, supports growth and defense, and a more responsive root system enhances resource capture while restricting Te mobility. This integrated model explains why the combined treatment produced broader recovery than either regulator alone, although the proposed causal links remain to be tested using transporter-expression analyses, ion-flux measurements, inhibitors, and genetic approaches.

**Figure 8.**
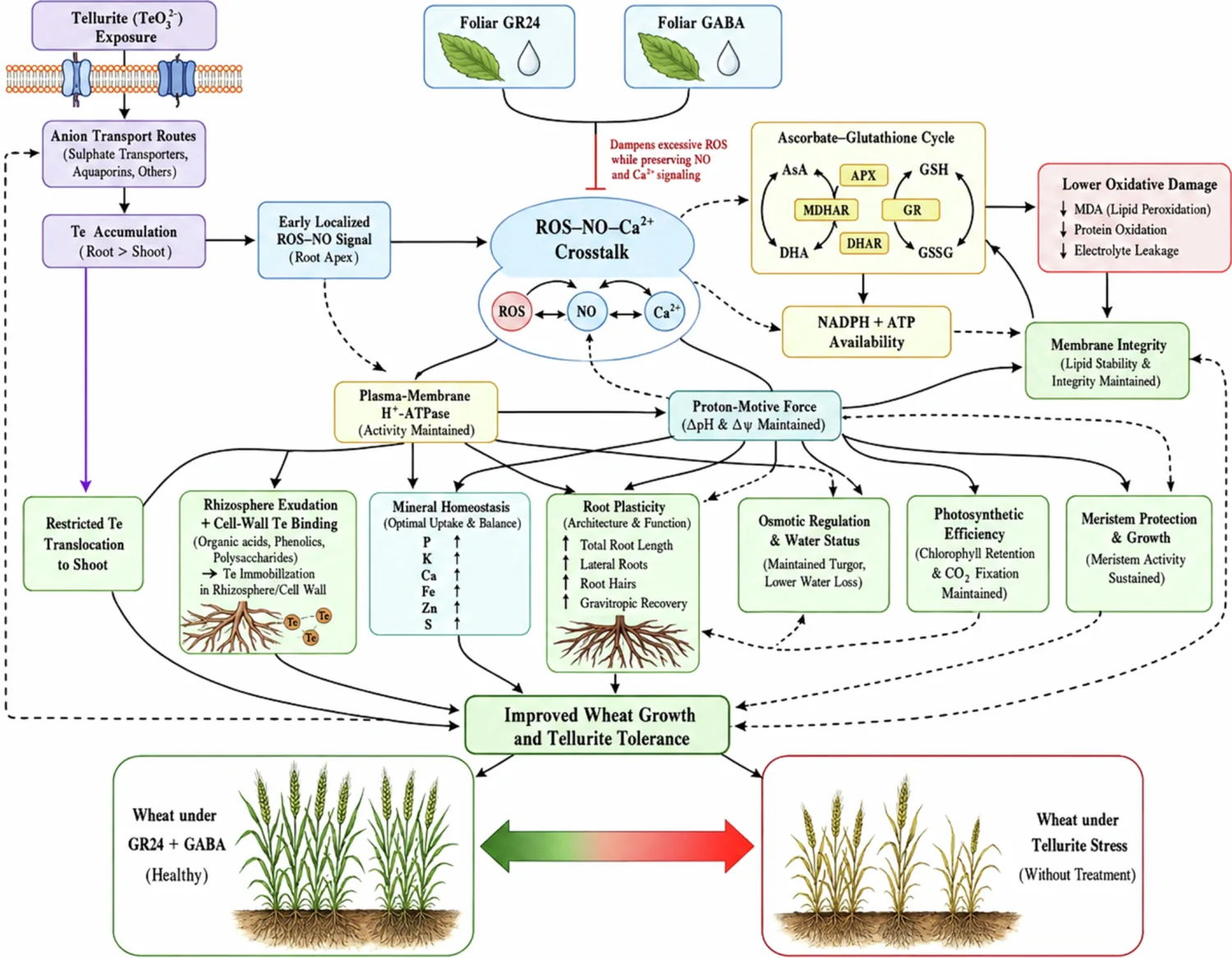
Proposed mechanistic model for the interaction of foliar GR24 and GABA in alleviating tellurite toxicity in wheat. GR24-associated strigolactone-auxin signaling and GABA-associated metabolic and anion-transport regulation are proposed to converge on ROS-NO-Ca^2^⁺ signal control, ascorbate-glutathione redox recovery, and plasma-membrane H⁺-ATPase energization. These coordinated responses support root-system remodeling, mineral/Te selectivity, rhizosphere regulation, and cell-wall Te sequestration, thereby restricting shoot Te translocation and improving stress tolerance. The solid arrows denote relationships supported by the measured physiological responses, whereas the dashed arrows denote literature-based molecular links that were not directly tested.

Based on the present findings and established signaling pathways, we propose that GR24 and GABA act through complementary but convergent routes, as summarized in Figure 8.

### 4.8. Practical implications, field feasibility, and limitations

Foliar delivery is agronomically attractive because it can be integrated with conventional crop-spraying equipment and avoids directly adding GR24 or GABA to contaminated soil, where either compound could be adsorbed, degraded, or alter tellurium speciation. The concentrations evaluated here were low (1  µM GR24 and 1  mM GABA), but the wire-house protocol used three applications and approximately 20  mL solution per plant until incipient run-off. Field translation should therefore optimize spray volume on an area basis, treatment timing, number of applications, surfactant concentration, and canopy coverage rather than directly extrapolating the per-plant volume used under controlled conditions. Its application during early stress development may be more efficient than repeated routine spraying, but this requires validation across wheat growth stages and under naturally occurring tellurium exposure.

Economic feasibility will depend largely on the GR24 component. GABA is a relatively simple metabolite that is commercially available and may be feasible for agricultural formulation, whereas GR24 is currently used mainly as a research-grade strigolactone analog, and its synthesis, purification, and limited bulk availability may make repeated field application comparatively expensive. Consequently, the combined treatment should not yet be considered a ready-to-use farm recommendation. Economic assessment should compare the cost of active ingredients, formulations, labor, and repeated spraying with the value of yield protection and any reduction in shoot or grain Te accumulation. Dose reduction, fewer applications, stable formulations, controlled-release carriers, or less expensive strigolactone analogs could improve cost-effectiveness. GR24 alone, GABA alone, and the combined treatment should also be compared in partial-budget and benefit-cost analyses to establish whether the additional benefit of the mixture justifies its additional cost.

Several factors may restrict field performance. Foliar uptake can vary with temperature, humidity, rainfall, solar radiation, leaf age, cultivar, spray-water quality, and canopy architecture, while photodegradation, wash-off, and limited cuticular penetration may shorten treatment persistence. The response may also depend on the soil texture, pH, organic matter, nutrient status, tellurium speciation, and the spatial heterogeneity of contamination. Moreover, the experimental tellurite dose exceeded the reported environmental concentrations, and physiological recovery does not by itself establish food-safety protection because the grain Te concentration, residue behavior, non-target effects, and environmental fate were not determined. Multi-location field trials using environmentally realistic Te levels are therefore required to identify the minimum effective dose, application window, and number of sprays; quantify the grain yield and grain Te; evaluate crop and soil safety; and determine the economic return before recommending GR24 + GABA for commercial wheat production.

## 5. Conclusion

Foliar application of GR24 and GABA, particularly in combination, modified the early redox and calcium-associated signaling response of wheat to tellurite, improved the GSH/GSSG and AsA/DHA ratios to a comparable degree, restored plasma-membrane H⁺-ATPase activity, enhanced root-system plasticity and gravitropic responsiveness, improved selectivity for all six measured mineral nutrients relative to tellurium, and increased tellurium retention in the root cell wall while restricting its translocation to the shoot. These findings identify signal regulation and membrane energization, rather than antioxidant enzyme induction alone, as the physiological basis of the observed tolerance and indicate that a foliar treatment can influence root-level behavior under a soil-borne oxyanion stress. Two limitations bound these conclusions. The treatments were characterized physiologically rather than molecularly, so the proposed sequence remains a hypothesis, and the tellurite dose used here was selected to resolve signaling events within one season and exceeds concentrations reported even in soils receiving electronic waste, so extrapolation to field contamination requires a dose‒response study at environmentally realistic concentrations.

## Data Availability

The datasets analyzed during this study are included in this manuscript.

## Statement on guidelines

All experimental studies and experimental materials involved in this research are in full compliance with relevant institutional, national, and international guidelines and legislation.

## References

[1] Belzile N , Chen Y-W . Tellurium in the environment: A critical review focused on natural waters, soils, sediments and airborne particles. ApGC. 2015;63:83–92. doi: 10.1016/j.apgeochem.2015.07.002.

[2] Zhang Q , Li Y , Fan X , Sun D , Sun J , Jiang X , Bai W . Cyanidin-3-glucoside-4-vinylphenol Mitigates Cadmium-Induced Hepatic and Colonic Injury via the Modulation of Lipid Metabolism and Gut Microbiota in Mice. J Agric Food Chem. 2026;74(31):24344–24360. doi: 10.1021/acs.jafc.6c03768. 42584145

[3] Grygoyć K , Jabłońska-Czapla M . Development of a tellurium speciation study using IC-ICP-MS on soil samples taken from an area associated with the storage, processing, and recovery of electrowaste. Molecules. 2021;26(9):2651. doi: 10.3390/molecules26092651. 33946621 PMC8124937

[4] Chasteen TG , Fuentes DE , Tantaleán JC , Vásquez CC . Tellurite: history, oxidative stress, and molecular mechanisms of resistance. FEMS Microbiol Rev. 2009;33(4):820–832. doi: 10.1111/j.1574-6976.2009.00177.x. 19368559

[5] Filella M , Reimann C , Biver M , Rodushkin I , Rodushkina K . Tellurium in the environment: current knowledge and identification of gaps. Environ Chem. 2019;16(4):215–228. doi: 10.1071/en18229.

[6] Turner RJ , Weiner JH , Taylor DE . Tellurite-mediated thiol oxidation in Escherichia coli. Microbiology. 1999;145(9):2549–2557. doi: 10.1099/00221287-145-9-2549. 10517608

[7] Turner RJ , Aharonowitz Y , Weiner JH , Taylor DE . Glutathione is a target in tellurite toxicity and is protected by tellurite resistance determinants in Escherichia coli. Can J Microbiol. 2001;47(1):33–40. doi: 10.1139/w00-125. 15049447

[8] Pérez JM , Calderón IL , Arenas FA , Fuentes DE , Pradenas GA , Sandoval JM , Castro ME , Elías AO , Vásquez CC , Sonenshein A . Bacterial toxicity of potassium tellurite: unveiling an ancient enigma. PLoS One. 2007;2(2):e211. doi: 10.1371/journal.pone.0000211. 17299591 PMC1784070

[9] Morales EH , Pinto CA , Luraschi R , Muñoz-Villagrán CM , Cornejo FA , Simpkins SW , Nelson J , Arenas FA , Piotrowski JS , Myers CL , et al. Accumulation of heme biosynthetic intermediates contributes to the antibacterial action of the metalloid tellurite. Nat Commun. 2017;8(1):15320. doi: 10.1038/ncomms15320. 28492282 PMC5437285

[10] Ottosson L-G , Logg K , Ibstedt S , Sunnerhagen P , Käll M , Blomberg A , Warringer J . Sulfate assimilation mediates tellurite reduction and toxicity in Saccharomyces cerevisiae. Eukaryot Cell. 2010;9(10):1635–1647. doi: 10.1128/ec.00078-10. 20675578 PMC2950436

[11] Cataldo D , Wildung R , Garland T . Speciation of trace inorganic contaminants in plants and bioavailability to animals: an overview. J Environ Qual. 1987;16(4):289–295. doi: 10.2134/jeq1987.00472425001600040001x.

[12] Takahashi H , Kopriva S , Giordano M , Saito K , Hell R . Sulfur assimilation in photosynthetic organisms: molecular functions and regulations of transporters and assimilatory enzymes. Annu Rev Plant Biol. 2011;62(1):157–184. doi: 10.1146/annurev-arplant-042110-103921. 21370978

[13] Mittler R , Zandalinas SI , Fichman Y , Van Breusegem F . Reactive oxygen species signalling in plant stress responses. Nat Rev Mol Cell Biol. 2022;23(10):663–679. doi: 10.1038/s41580-022-00499-2. 35760900

[14] Foyer CH , Noctor G . Redox homeostasis and antioxidant signaling: a metabolic interface between stress perception and physiological responses. Plant Cell. 2005;17(7):1866–1875. doi: 10.1105/tpc.105.033589. 15987996 PMC1167537

[15] Bienert GP , Chaumont F . Aquaporin-facilitated transmembrane diffusion of hydrogen peroxide. Biochimica et Biophysica Acta (BBA)-General Subjects. 2014;1840(5):1596–1604. doi: 10.1016/j.bbagen.2013.09.017. 24060746

[16] Suzuki N , Miller G , Morales J , Shulaev V , Torres MA , Mittler R . Respiratory burst oxidases: the engines of ROS signaling. Curr Opin Plant Biol. 2011;14(6):691–699. doi: 10.1016/j.pbi.2011.07.014. 21862390

[17] Choudhury FK , Rivero RM , Blumwald E , Mittler R . Reactive oxygen species, abiotic stress and stress combination. Plant J. 2017;90(5):856–867. doi: 10.1111/tpj.13299. 27801967

[18] Khator K , Parihar S , Jasik J , Shekhawat GS . Nitric oxide in plants: an insight on redox activity and responses toward abiotic stress signaling. Plant Signaling & Behavior. 2024;19(1):2298053. doi: 10.1080/15592324.2023.2298053. 38190763 PMC10793691

[19] Kolbert Z , Feigl G , Freschi L , Poór P . Gasotransmitters in action: nitric oxide-ethylene crosstalk during plant growth and abiotic stress responses. Antioxidants. 2019;8(6):167. doi: 10.3390/antiox8060167. 31181724 PMC6616412

[20] Zhou X , Joshi S , Patil S , Khare T , Kumar V . Reactive oxygen, nitrogen, carbonyl and sulfur species and their roles in plant abiotic stress responses and tolerance. J Plant Growth Regul. 2022;41(1):119–142. doi: 10.1007/s00344-020-10294-y.

[21] Kudla J , Becker D , Grill E , Hedrich R , Hippler M , Kummer U , Parniske M , Romeis T , Schumacher K . Advances and current challenges in calcium signaling. New Phytol. 2018;218(2):414–431. doi: 10.1111/nph.14966. 29332310

[22] Marcec MJ , Gilroy S , Poovaiah B , Tanaka K . Mutual interplay of Ca2+ and ROS signaling in plant immune response. Plant Sci. 2019;283:343–354. doi: 10.1016/j.plantsci.2019.03.004. 31128705

[23] Choi W-G , Toyota M , Kim S-H , Hilleary R , Gilroy S . Salt stress-induced Ca2+ waves are associated with rapid, long-distance root-to-shoot signaling in plants. Proc Natl Acad Sci. 2014;111(17):6497–6502. doi: 10.1073/pnas.1319955111. 24706854 PMC4035928

[24] Waters MT , Gutjahr C , Bennett T , Nelson DC . Strigolactone signaling and evolution. Annu Rev Plant Biol. 2017;68:291–322. doi: 10.1146/annurev-arplant-042916-040925. 28125281

[25] Ruyter-Spira C , Kohlen W , Charnikhova T , van Zeijl A , van Bezouwen L , de Ruijter N , Cardoso C , Lopez-Raez JA , Matusova R , Bours R , et al. Physiological effects of the synthetic strigolactone analog GR24 on root system architecture in Arabidopsis: another belowground role for strigolactones? Plant Physiol. 2011;155(2):721–734. doi: 10.1104/pp.110.166645. 21119044 PMC3032462

[26] Kapulnik Y , Delaux P-M , Resnick N , Mayzlish-Gati E , Wininger S , Bhattacharya C , Séjalon-Delmas N , Combier J , Bécard G , Belausov E , et al. Strigolactones affect lateral root formation and root-hair elongation in Arabidopsis. Planta. 2011;233(1):209–216. doi: 10.1007/s00425-010-1310-y. 21080198

[27] Trasoletti M , Visentin I , Campo E , Schubert A , Cardinale F . Strigolactones as a hormonal hub for the acclimation and priming to environmental stress in plants. Plant, Cell & Environment. 2022;45(12):3611–3630. doi: 10.1111/pce.14461. PMC9828678 36207810

[28] Kohlen W , Charnikhova T , Liu Q , Bours R , Domagalska MA , Beguerie S , Verstappen F , Leyser O , Bouwmeester H , Ruyter-Spira C . Strigolactones are transported through the xylem and play a key role in shoot architectural response to phosphate deficiency in nonarbuscular mycorrhizal host Arabidopsis. Plant Physiol. 2011;155(2):974–987. doi: 10.1104/pp.110.164640. 21119045 PMC3032481

[29] Ramesh SA , Tyerman SD , Gilliham M , Xu B . γ-Aminobutyric acid (GABA) signalling in plants. Cell Mol Life Sci. 2017;74(9):1577–1603. doi: 10.1007/s00018-016-2415-7. 27838745 PMC11107511

[30] Ramesh SA , Tyerman SD , Xu B , Bose J , Kaur S , Conn V , Domingos P , Ullah S , Wege S , Shabala S , et al. GABA signalling modulates plant growth by directly regulating the activity of plant-specific anion transporters. Nat Commun. 2015;6(1):7879. doi: 10.1038/ncomms8879. 26219411 PMC4532832

[31] Xu B , Long Y , Feng X , Zhu X , Sai N , Chirkova L , Betts A , Herrmann J , Edwards EJ , Okamoto M , et al. GABA signalling modulates stomatal opening to enhance plant water use efficiency and drought resilience. Nat Commun. 2021;12(1):1952. doi: 10.1038/s41467-021-21694-3. 33782393 PMC8007581

[32] Bouyoucos GJ . Hydrometer method improved for making particle size analyses of soils 1. Agron J. 1962;54(5):464–465. doi: 10.2134/agronj1962.00021962005400050028x.

[33] Walkley A , Black IA . An examination of the Degtjareff method for determining soil organic matter, and a proposed modification of the chromic acid titration method. Soil Sci. 1934;37(1):29–38. doi: 10.1097/00010694-193401000-00003.

[34] Olsen SR . Estimation of available phosphorus in soils by extraction with sodium bicarbonate. US Department of Agriculture. 1954.

[35] Lindsay WL , Norvell WA . Development of a DTPA soil test for zinc, iron, manganese, and copper. Soil Sci Am J. 1978;42(3):421–428. doi: 10.2136/sssaj1978.03615995004200030009x.

[36] Qiu C-W , Zhang C , Wang N-H , Mao W , Wu F . Strigolactone GR24 improves cadmium tolerance by regulating cadmium uptake, nitric oxide signaling and antioxidant metabolism in barley (Hordeum vulgare L. Environ Pollut. 2021;273:116486. doi: 10.1016/j.envpol.2021.116486. 33484996

[37] Zhao Q , Ma Y , Huang X , Song L , Li N , Qiao M , Hai D , Cheng Y . GABA application enhances drought stress tolerance in wheat seedlings (Triticum aestivum L. Plants. 2023;12(13):2495. doi: 10.3390/plants12132495. 37447056 PMC10346274

[38] Velikova V , Yordanov I , Edreva A . Oxidative stress and some antioxidant systems in acid rain-treated bean plants: protective role of exogenous polyamines. Plant Sci. 2000;151(1):59–66. doi: 10.1016/S0168-9452(99)00197-1.

[39] Elstner EF , Heupel A . Inhibition of nitrite formation from hydroxylammoniumchloride: a simple assay for superoxide dismutase. Anal Biochem. 1976;70(2):616–620. doi: 10.1016/0003-2697(76)90488-7. 817618

[40] Zhou B , Guo Z , Xing J , Huang B . Nitric oxide is involved in abscisic acid-induced antioxidant activities in Stylosanthes guianensis. J Exp Bot. 2005;56(422):3223–3228. doi: 10.1093/jxb/eri319. 16263901

[41] Sagi M , Fluhr R . Superoxide production by plant homologues of the gp91phox NADPH oxidase. Modulation of activity by calcium and by tobacco mosaic virus infection. Plant Physiol. 2001;126(3):1281–1290. doi: 10.1104/pp.126.3.1281. 11457979 PMC116485

[42] Collinge D . Subcellular localization of H2O2 in plants. H2O2 accumulation in papillae and hypersensitive response during the barley-powdery mildew interaction. Plant J. 1997;11:1187–1194. doi: 10.1046/j.1365-313X.1997.11061187.x.

[43] Dunand C , Crèvecoeur M , Penel C . Distribution of superoxide and hydrogen peroxide in Arabidopsis root and their influence on root development: possible interaction with peroxidases. New Phytol. 2007;174(2):332–341. doi: 10.1111/j.1469-8137.2007.01995.x. 17388896

[44] Rodríguez-Serrano M , Romero-Puertas MC , Pazmiño DM , Testillano PS , Risueño MC , Del Río LA , Sandalio LM , et al. Cellular response of pea plants to cadmium toxicity: cross talk between reactive oxygen species, nitric oxide, and calcium. Plant Physiol. 2009;150(1):229–43. doi: 10.1104/pp.108.131524. 19279198 PMC2675729

[45] Kiegle E , Moore CA , Haseloff J , Tester MA , Knight MR . Cell‐type‐specific calcium responses to drought, salt and cold in the Arabidopsis root. Plant J. 2000;23(2):267–278. doi: 10.1046/j.1365-313x.2000.00786.x. 10929120

[46] Himmelbauer ML , Loiskandl AW , Kastanek AF . Estimating length, average diameter and surface area of roots using two different image analyses systems. Plant Soil. 2004;260(1):111–120. doi: 10.1023/b:plso.0000030171.28821.55.

[47] Nagel KA , Putz A , Gilmer F , Heinz K , Fischbach A , Pfeifer J , Faget M , Blossfeld S , Ernst M , Dimaki C , et al. GROWSCREEN-Rhizo is a novel phenotyping robot enabling simultaneous measurements of root and shoot growth for plants grown in soil-filled rhizotrons. Funct Plant Biol. 2012;39(11):891–904. doi: 10.1071/fp12023. 32480839

[48] Bates T , Lynch J . Stimulation of root hair elongation in Arabidopsis thaliana by low phosphorus availability. Plant, cell & environment. 1996;19(5):529–538. doi: 10.1111/j.1365-3040.1996.tb00386.x.

[49] Grabov A , Ashley M , Rigas S , Hatzopoulos P , Dolan L , Vicente‐Agullo F . Morphometric analysis of root shape. New Phytol. 2005;165(2):641–652. doi: 10.1111/j.1469-8137.2004.01258.x. 15720674

[50] Steponkus PL , Lanphear F . Refinement of the triphenyl tetrazolium chloride method of determining cold injury. Plant Physiol. 1967;42(10):1423–1426. doi: 10.1104/pp.42.10.1423. 16656672 PMC1086741

[51] Jacyn Baker C , Mock NM . An improved method for monitoring cell death in cell suspension and leaf disc assays using Evans blue. Plant Cell, Tissue Organ Cult. 1994;39(1):7–12. doi: 10.1007/bf00037585.

[52] Heath RL , Packer L . Photoperoxidation in isolated chloroplasts: I. Kinetics and stoichiometry of fatty acid peroxidation. Arch Biochem Biophys. 1968;125(1):189–198. doi: 10.1016/0003-9861(68)90654-1. 5655425

[53] Levine RL , Garland D , Oliver CN , Amici A , Climent I , Lenz A , Ahn B , Shaltiel S , Stadtman ER . [49] Determination of carbonyl content in oxidatively modified proteins. Methods Enzymol. Vol. 186 Elsevier; 1990; p. 464–478. doi: 10.1016/0076-6879(90)86141-H. 1978225

[54] Sairam R , Deshmukh P , Shukla D . Tolerance of drought and temperature stress in relation to increased antioxidant enzyme activity in wheat. J. Agron. Crop Sci. 1997;178(3):171–178. doi: 10.1111/j.1439-037x.1997.tb00486.x.

[55] Giannopolitis CN , Ries SK . Superoxide dismutases: I. Occurrence in higher plants. Plant Physiol. 1977;59(2):309–314. doi: 10.1104/pp.59.2.309. 16659839 PMC542387

[56] Aebi H . [13] Catalase in vitro. Methods Enzymol. Vol. 105 Elsevier; 1984; p. 121–126. doi: 10.1016/S0076-6879(84)05016-3. 6727660

[57] Nakano Y , Asada K . Hydrogen peroxide is scavenged by ascorbate-specific peroxidase in spinach chloroplasts. Plant Cell Physiol. 1981;22(5):867–880. doi: 10.1093/oxfordjournals.pcp.a076232.

[58] Foyer CH , Halliwell B . The presence of glutathione and glutathione reductase in chloroplasts: a proposed role in ascorbic acid metabolism. Planta. 1976;133(1):21–25. doi: 10.1007/bf00386001. 24425174

[59] Law M , Charles SA , Halliwell B . Glutathione and ascorbic acid in spinach (Spinacia oleracea) chloroplasts. The effect of hydrogen peroxide and of paraquat. Biochem J. 1983;210(3):899–903. doi: 10.1042/bj2100899. 6307273 PMC1154305

[60] Griffith OW . Determination of glutathione and glutathione disulfide using glutathione reductase and 2-vinylpyridine. Anal Biochem. 1980;106(1):207–212. doi: 10.1016/0003-2697(80)90139-6. 7416462

[61] Schafer FQ , Buettner GR . Redox environment of the cell as viewed through the redox state of the glutathione disulfide/glutathione couple. Free Radic Biol Med. 2001;30(11):1191–1212. doi: 10.1016/s0891-5849(01)00480-4. 11368918

[62] Queval G , Noctor G . A plate reader method for the measurement of NAD, NADP, glutathione, and ascorbate in tissue extracts: application to redox profiling during Arabidopsis rosette development. Anal Biochem. 2007;363(1):58–69. doi: 10.1016/j.ab.2007.01.005. 17288982

[63] Lundin A . Use of firefly luciferase in ATP-related assays of biomass, enzymes, and metabolites (6879 2000; p. 05499–05499 00). 10.1016/s0076-6879(00)05499-9 10812612

[64] Palmgren MG . An H+-ATPase assay: proton pumping and ATPase activity determined simultaneously in the same sample. Plant Physiol. 1990;94(3):882–886. doi: 10.1104/pp.94.3.882. 16667867 PMC1077317

[65] Yan F , Feuerle R , Schäffer S , Fortmeier H , Schubert S . Adaptation of active proton pumping and plasmalemma ATPase activity of corn roots to low root medium pH. Plant Physiol. 1998;117(1):311–319. doi: 10.1104/pp.117.1.311. 9576801 PMC35017

[66] Tabatabai MA , Bremner JM . Use of p-nitrophenyl phosphate for assay of soil phosphatase activity. Soil Biol Biochem. 1969;1(4):301–307. doi: 10.1016/0038-0717(69)90012-1.

[67] Neumann G , Romheld V . The release of root exudates as affected by the plant's physiological status. The rhizosphere. CRC press; 2000; p. 57–110.

[68] Hansen T , De Bang T , Laursen K , et al. Multielement plant tissue analysis using ICP spectrometry. In Plant mineral nutrients: Methods and protocols. 2012. pp. 121–141 Springer. 10.1007/978-1-62703-152-3_8 23073880

[69] Weigel HJ , Jäger HJ . Subcellular distribution and chemical form of cadmium in bean plants. Plant Physiol. 1980;65(3):480–482. doi: 10.1104/pp.65.3.480. 16661218 PMC440359

[70] Von Caemmerer S , Farquhar GD . Some relationships between the biochemistry of photosynthesis and the gas exchange of leaves. Planta. 1981;153(4):376–387. doi: 10.1007/bf00384257. 24276943

[71] Kitajima M , Butler W . Quenching of chlorophyll fluorescence and primary photochemistry in chloroplasts by dibromothymoquinone. Biochimica et Biophysica Acta (BBA)-Bioenergetics. 1975;376(1):105–115. doi: 10.1016/0005-2728(75)90209-1. 1125215

[72] Genty B , Briantais J-M , Baker NR . The relationship between the quantum yield of photosynthetic electron transport and quenching of chlorophyll fluorescence. Biochimica et biophysica acta (BBA)-General subjects. 1989;990(1):87–92. doi: 10.1016/s0304-4165(89)80016-9.

[73] Bilger W , Björkman O . Role of the xanthophyll cycle in photoprotection elucidated by measurements of light-induced absorbance changes, fluorescence and photosynthesis in leaves of Hedera canariensis. Photosynth Res. 1990;25(3):173–185. doi: 10.1007/BF00033159. 24420348

[74] Barrs H , Weatherley P . A re-examination of the relative turgidity technique for estimating water deficits in leaves. Aust J Biol Sci. 1962;15(3):413–428. doi: 10.1071/bi9620413.

[75] Sun C , Lu L , Liu L , Yu Y , Hu Y , Jin C , Lin X . Nitrate reductase‐mediated early nitric oxide burst alleviates oxidative damage induced by aluminum through enhancement of antioxidant defenses in roots of wheat (Triticum aestivum). New Phytol. 2014;201(4):1240–1250. doi: 10.1111/nph.12597. 24237306

[76] Catarecha P , Segura MD , Franco-Zorrilla JM , García-Ponce B , Lanza M , Solano R , Paz-Ares J , Leyva A . A mutant of the Arabidopsis phosphate transporter PHT1; 1 displays enhanced arsenic accumulation. Plant Cell. 2007;19(3):1123–1133. doi: 10.1105/tpc.106.041871. 17400898 PMC1867351

[77] Abercrombie JM , Halfhill MD , Ranjan P , Rao MR , Saxton AM , Yuan JS , Stewart CN . Transcriptional responses of Arabidopsis thaliana plants to As (V) stress. BMC Plant Biol. 2008;8(1):87. doi: 10.1186/1471-2229-8-87. 18684332 PMC2547109

[78] Xu Z-R , Cai M-L , Chen S-H , Huang X , Zhao F , Wang P . High-affinity sulfate transporter Sultr1; 2 is a major transporter for Cr (VI) uptake in plants. Environ Sci Technol. 2021;55(3):1576–1584. doi: 10.1021/acs.est.0c04384. 33423475

[79] Tai Z , Yin X , Fang Z , Shi G , Lou L , Cai Q . Exogenous GR24 alleviates cadmium toxicity by reducing cadmium uptake in switchgrass (Panicum virgatum) seedlings. Int J Environ Res Public Health. 2017;14(8):852. doi: 10.3390/ijerph14080852. 28758909 PMC5580556

[80] Buchcik W , Sitko K , Marzec M . Dissecting the role of strigolactone perception in barley tolerance to cadmium or zinc stress based on the receptor mutant analysis. BMC Plant Biol. 2025;25(1):1618. doi: 10.1186/s12870-025-07683-4. 41272468 PMC12639677

[81] Sedaghat M , Tahmasebi-Sarvestani Z , Emam Y , Mokhtassi-Bidgoli A . Physiological and antioxidant responses of winter wheat cultivars to strigolactone and salicylic acid in drought. Plant Physiol Biochem. 2017;119:59–69. doi: 10.1016/j.plaphy.2017.08.015. 28843889

[82] Sedaghat M , Emam Y , Mokhtassi-Bidgoli A , Hazrati S , Lovisolo C , Visentin I , Cardinale F , Tahmasebi-Sarvestani Z . The potential of the synthetic strigolactone analogue GR24 for the maintenance of photosynthesis and yield in winter wheat under drought: investigations on the mechanisms of action and delivery modes. Plants. 2021;10(6):1223. doi: 10.3390/plants10061223. 34208497 PMC8233996

[83] Ahmad S , Fariduddin Q . Deciphering the enigmatic role of gamma-aminobutyric acid (GABA) in plants: Synthesis, transport, regulation, signaling, and biological roles in interaction with growth regulators and abiotic stresses. Plant Physiol Biochem. 2024;208:108502. doi: 10.1016/j.plaphy.2024.108502. 38492486

[84] Foyer CH , Noctor G . Ascorbate and glutathione: the heart of the redox hub. Plant Physiol. 2011;155(1):2–18. doi: 10.1104/pp.110.167569. 21205630 PMC3075780

[85] Noctor G , Mhamdi A , Chaouch S , HAN Y , NEUKERMANS J , MARQUEZ‐GARCIA B , QUEVAL G , FOYER CH . Glutathione in plants: an integrated overview. Plant, cell & environment. 2012;35(2):454–484. doi: 10.1111/j.1365-3040.2011.02400.x. 21777251

[86] Falhof J , Pedersen JT , Fuglsang AT , Palmgren M . Plasma membrane H+-ATPase regulation in the center of plant physiology. Mol Plant. 2016;9(3):323–337. doi: 10.1016/j.molp.2015.11.002. 26584714

[87] Zeng H , Chen H , Zhang M , Ding M , Xu F , Yan F , Kinoshita T , Zhu Y . Plasma membrane H+-ATPases in mineral nutrition and crop improvement. Trends Plant Sci. 2024;29(9):978–994. doi: 10.1016/j.tplants.2024.02.010. 38582687

[88] Janicka-Russak M , Kabała K , Burzyński M , Kabala K , Burzynski M , Klobus G . Response of plasma membrane H+-ATPase to heavy metal stress in Cucumis sativu s roots. J Exp Bot. 2008;59(13):3721–3728. doi: 10.1093/jxb/ern219. 18820260 PMC2561156

[89] Ryan P , Delhaize E , Jones D . Function and mechanism of organic anion exudation from plant roots. Annu Rev Plant Biol. 2001;52(1):527–560. doi: 10.1146/annurev.arplant.52.1.527. 11337408

[90] Long Y , Tyerman SD , Gilliham M . Cytosolic GABA inhibits anion transport by wheat ALMT1. New Phytol. 2020;225(2):671–678. doi: 10.1111/nph.16238. 31591723

[91] Ramesh SA , Kamran M , Sullivan W , Chirkova L , Okamoto M , Degryse F , McLaughlin M , Gilliham M , Tyerman SD . Aluminum-activated malate transporters can facilitate GABA transport. Plant Cell. 2018;30(5):1147–1164. doi: 10.1105/tpc.17.00864. 29618628 PMC6002190

[92] Hall . Já. Cellular mechanisms for heavy metal detoxification and tolerance. J Exp Bot. 2002;53(366):1–11. doi: 10.1093/jexbot/53.366.1. 11741035

[93] Krzesłowska M . The cell wall in plant cell response to trace metals: polysaccharide remodeling and its role in defense strategy. Acta Physiol Plant. 2011;33(1):35–51. doi: 10.1007/s11738-010-0581-z.

